# On-demand switching from mono-silylene to bis-silylene to access mono-, di- and mixed coinage metal complexes

**DOI:** 10.1039/d5sc04287a

**Published:** 2025-08-15

**Authors:** Xiaofei Sun, Da Jin, Ravi Yadav, Frederic Kraetschmer, Ralf Köppe, Peter W. Roesky

**Affiliations:** a Institute of Inorganic Chemistry (AOC), Karlsruhe Institute of Technology (KIT) Kaiserstr. 12 Karlsruhe 76131 Germany roesky@kit.edu; b School of Chemistry, Indian Institute of Science Education and Research Thiruvananthapuram Thiruvananthapuram-695551 Kerala India; c Institute of Nanotechnology (INT), Karlsruhe Institute of Technology (KIT) Kaiserstr. 12 Karlsruhe 76131 Germany

## Abstract

Recently, we discovered that silaiminyl-silylene, [LSi–Si(NDipp)L] (L = PhC(N*t*Bu)_2_, Dipp = 2,6-diisopropylphenyl), can be converted from a mono-silylene to bis-silylene by using Lewis acids. This revelation led us to further use silaiminyl-silylene as a silylene-based ligand, which can coordinate to one metal center and later, on demand, release one more silylene center to coordinate to a second metal. Furthermore, an insight into the mechanism of this unusual rearrangement reaction is presented. Initially, mono-silylene complexes [LSi{M(Mes)}–Si(NDipp)L] (M = Ag, Au) were isolated. These complexes were then used as templates to access bis-silylene coordinated homo-dinuclear, [LSi{M(Mes)}–(NDipp)–{M(Mes)}SiL] (M = Ag, Au) and hetero-dinuclear, [LSi{Ag(Mes)}–(NDipp)–{Au(Mes)}SiL] complexes *via* a Lewis acid triggered ligand rearrangement. Notably, using this silylene, a selective coordination of the two different coinage metals Ag and Au was achieved stepwise. This differs from the reactivity when a conventional bis-silylene is employed. The isolation of [LSi{Ag(Mes)}–(NDipp)–{Au(Mes)}SiL] showcases the utility of strong σ-donor silylene-based switchable ligands. This represents the first example of a heterobimetallic complex ligated by a spacer-separated bis-silylene ligand.

## Abstract 2

Investigations into the reactivity and rearrangement of a silaiminyl-silylene with suitable Ag and Au precursors in different stochiometric ratios have unveiled intriguing insights into its dynamic behavior and capability to act as a silylene ligand.

## Graphical abstract

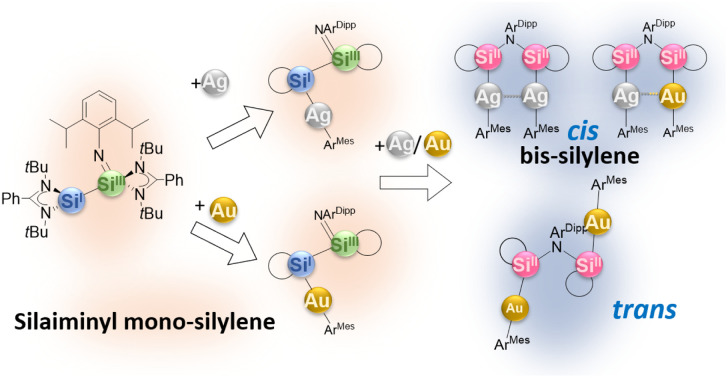

## Introduction

**Scheme 1:**
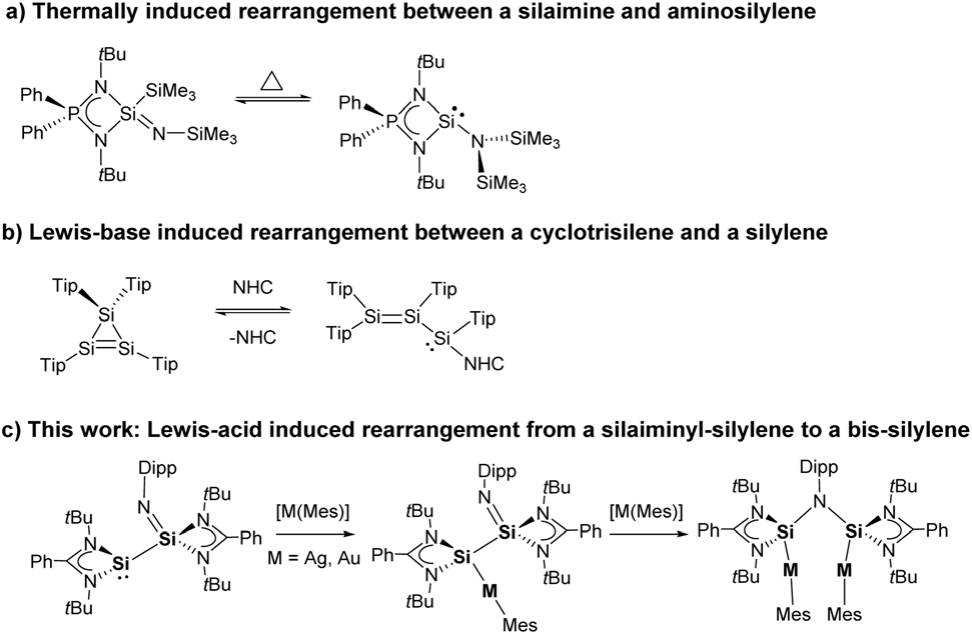
Selected examples of rearrangement reactions in silylene chemistry due to external stimuli (Tip = 2,4,6-triisopropylphenyl).^15,16^

Silylene chemistry has undergone immense advances since the initial reports of the silicocene^1^ and the *N*-heterocyclic silylene.^2^ Over time, with the help of the kinetic and/or the thermodynamic stabilization, a plethora of stable silylenes have been isolated.^3^ These compounds generally serve as strong donating ligands, and their transition metal complexes have found applications in homogeneous catalysis.^4–12^ Owing to their high reactivity, silylenes and their respective complexes have facilitated activation of diverse small molecules.^13^ More recently, the synthesis of metallylones has been achieved with the help of silylenes.^14^ Furthermore, rearrangement reactions in silylene chemistry triggered by external stimuli have emerged as an intriguing area of synthetic chemistry. In general, stimuli include, for example, temperature, pressure, solvent, light, and Lewis acids or bases. Upon the application of such a stimulus, the geometry and connectivity of the molecule can be changed. While stimuli-responsive materials, in particular polymers, have gained significant attention for their ability to alter properties in response to external stimuli, stimuli-responsive silylenes remain relatively scarce. Nevertheless, a few examples have been documented in the literature.^15–20^ Very recently, Nakata reported the interconversion between an iminophosphonamide-supported silaimine and aminosilylene by varying the temperature or using an oxidizing agent (Scheme 1a).^15^ Another example by Scheschkewitz and co-workers demonstrated that the addition of a Lewis base, an *N*-heterocyclic carbene (NHC), to a cyclotrisilene resulted in rearrangement to a disilenyl-silylene *via* Si–Si bond cleavage and coordination of the NHC fragment to the silylene.^16^ Recently, our group has reported the high yield access of a mixed-valent silaiminyl-silylene [LSi–Si(NDipp)L] (L = PhC(N*t*Bu)_2_, Dipp = 2,6-diisopropylphenyl),^19^ which rearranged upon treatment with an equimolar amount of copper-halide or mesityl-copper to form symmetric bis-silylene copper complexes, in which the Cu centers are in a trigonal planar coordination environment.

We postulated that the mechanism for the interconversion proceeds through stepwise coordination of Cu(i) to silaiminyl-silylene and then switching to bis-silylene. However, in the case of Cu(i), no such intermediate could be isolated or detected due to the highly exergonic reaction. This motivated us to investigate the proposed mechanism with Ag(i) and Au(i) to isolate the proposed intermediates. In this work, we report a stepwise, Lewis acid-induced rearrangement from a silaiminyl-silylene to a bis-silylene by using the heavier silver- or gold-based Lewis acids. The stimuli-responsive silaiminyl-silylene described herein acts as a “masked” bis-silylene. This stepwise metalation of the ligand not only allows isolation of M_2_ (M = Ag, Au) type complexes but also of a Ag–Au complex, which is otherwise impossible in the case of rigid bis-silylene ligands.

## Results and discussion

**Scheme 2:**
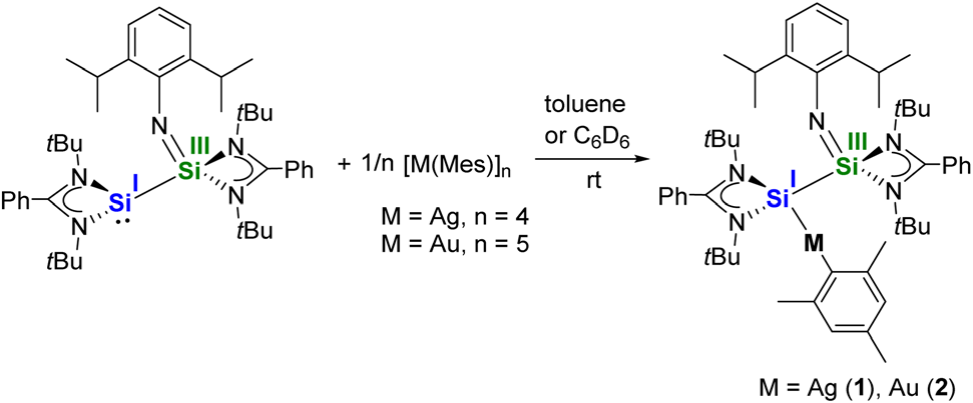
Synthesis of the silaiminyl-silylene Ag and Au complexes [LSi{Ag(Mes)}–Si(NDipp)L] (1) and [LSi{Au(Mes)}–Si(NDipp)L] (2).

**Fig. 1:**
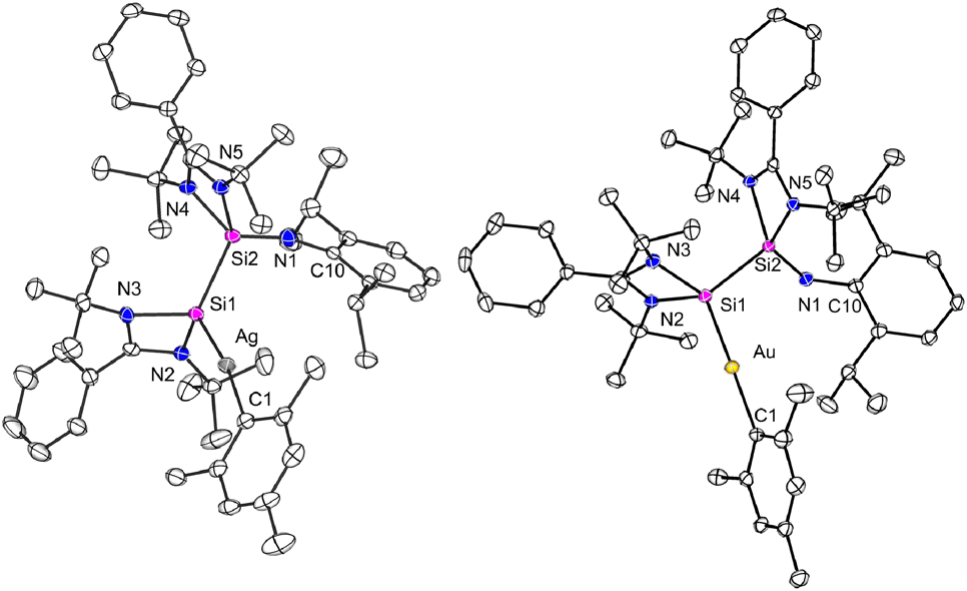
Molecular structures of complexes 1 and 2 in the solid state. All hydrogen atoms are omitted for clarity. Selected bond distances [Å] and angles [°] in 1: Ag–Si1 2.3847(5), Ag–C1 2.122(2), Si1–Si2 2.3593(7), Si2–N1 1.576(2), N1–C10 1.368(2); Si1–Ag–C1 169.84(5), Ag–Si1–Si2 125.16(2), Si1–Si2–N1 117.28(6), Si2–N1–C10 164.81(14). In 2: Au–Si1 2.3006(8), Au–C1 2.079(3), Si1–Si2 2.3514(11), Si2–N1 1.597(3), N1–C10 1.385(4); Si1–Au–C1 175.21(9), Au–Si1–Si2 114.49(4), Si1–Si2–N1 109.75(10), Si2–N1–C10 143.2(2).

The silaiminyl-silylene [LSi–Si(NDipp)L]^19^ was reacted with equimolar amounts of mesityl-silver and mesityl-gold, which afforded the silaiminyl-silylene Ag and Au complexes [LSi{M(Mes)}–Si(NDipp)L] (M = Ag (1), Au (2)) (Scheme 2) as yellow crystals in 51% and 47% yields, respectively. Single-crystal X-ray diffraction (SCXRD) analysis confirmed the molecular structures of compounds 1 and 2, revealing the formation of silaiminyl-silylene coinage metal complexes in which the coinage metal centers are coordinated by only one silicon atom and one mesityl ligand in a linear fashion (Fig. 1). The Si1–M–C1 angle for the Au complex 2 is relatively more linear (175.21(9)°) than that for the Ag complex 1 (169.84(5)°). Both Si atoms in complexes 1 and 2 are four-coordinate with distorted tetrahedral geometry. From Ag to Au, the Si–M bond distances decrease from 2.3847(5) Å (1) to 2.3006(8) Å (2); these values lie within the range of previously reported coinage metal silylene complexes (Ag: 2.337–2.425 Å; Au: 2.246–2.318 Å).^21–24^ A comparable trend (Si–Au < Si–Ag) has been previously observed in the work of Khan and Inoue.^21,25^ Upon coordination of the silylene to the coinage metal (Ag and Au) moieties, the silaiminyl-silylene ligand framework remains almost unchanged. Therefore, the formal oxidation states of the Si atoms could still be defined as +I and +III. Interestingly, the reactivity of silaiminyl-silylene with mesityl-silver and mesityl-gold differs from that with mesityl-copper.^19^ Unlike the former, a symmetrical bis-silylene-copper complex was formed, with the copper center adopting a trigonal planar geometry.

Despite postulations suggesting the formation of a linearly coordinated Cu silaiminyl-silylene complex as an intermediate, no experimental observation could support this hypothesis. Using the heavier Ag and Au precursors, stable analogs of this postulated intermediate were isolated.

The NMR spectra of both complexes were recorded in C_6_D_6_. The ^1^H NMR spectra for both silylene complexes 1 and 2 are comparable. In addition to the signals for the mesityl, phenyl and Dipp groups, each displays two singlet signals for the 36 protons of the four *t*Bu groups, which is consistent with the inequivalency of the two amidinate moieties. The ^29^Si{^1^H} NMR spectrum of the Ag silylene complex 1 displays two doublets for the silylene ^29^Si nuclei at 48.5 ppm, corresponding to the coupling with ^107^Ag (^1^*J*_29Si_–_107Ag_ = 188 Hz) and ^109^Ag (^1^*J*_29Si_–_109Ag_ = 217 Hz) isotopes, and one doublet for the silaiminyl ^29^Si nuclei at −72.4 ppm (^2^*J*_29Si–107/109Ag_ = 28 Hz). The coordination of the silylene to the Ag center is accompanied by a downfield shift for the silylene ^29^Si nuclei from 31.8 ppm to 48.5 ppm and a highfield shift for the silaimine ^29^Si nuclei from −61.7 ppm to −72.4 ppm.^19^ The silaimine signal is in the range of typical silaimines (−20 ppm to −105 ppm).^26–29^ Complex 2 exhibited poor solubility in C_6_D_6_. The ^13^C{^1^H} NMR spectrum of 2 does not show all resonances, and no ^29^Si signal could be detected. Both complexes were not stable in solution (C_6_D_6_), and decomposition took place during the course of several days.

**Scheme 3:**
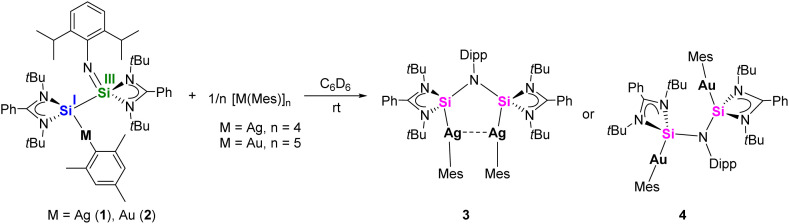
Synthesis of the bis-silylene Ag and Au complexes [LSi{Ag(Mes)}–(NDipp)–{Ag(Mes)}SiL] (3) and [LSi{Au(Mes)}–(NDipp)–{Au(Mes)}SiL] (4).

**Fig. 2:**
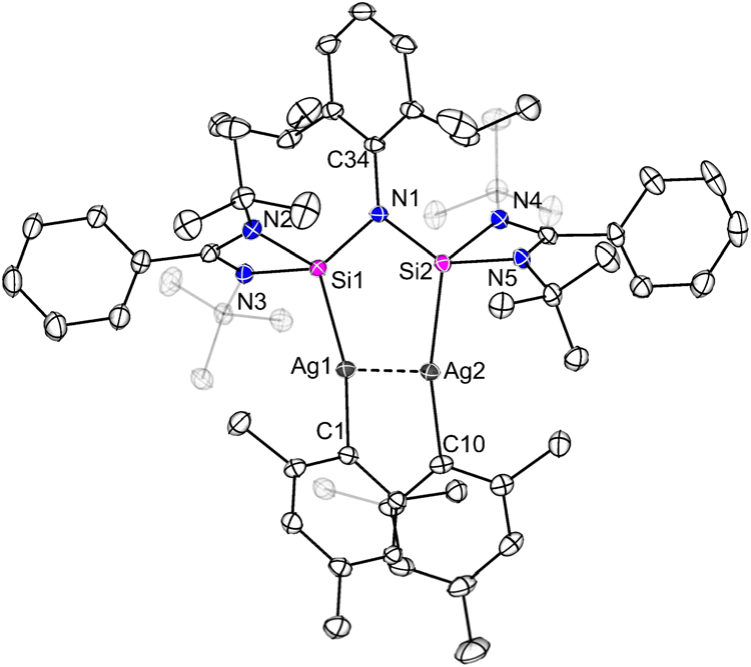
Molecular structure of complex 3 in the solid state. All hydrogen atoms and non-coordinating solvents are omitted for clarity. Selected bond distances [Å] and angles [°]: Ag1⋯Ag2 3.1235(3), Ag1–Si1 2.3993(5), Ag2–Si2 2.4191(5), Ag1–C1 2.135(2), Ag1–C10 2.147(2), Si1–N1 1.7741(13), Si2–N1 1.7796(14), N1–C34 1.471(2); Si1–Ag1–C1 164.11(5), Si2–Ag2–C2 165.52(5), Si1–N1–Si2 104.35(7).

To induce the ligand rearrangement, [AgMes]_4_ was added to a C_6_D_6_ solution of the Ag complex 1 (Scheme 3). The color of the solution turned from orange-red to light-orange, and after concentration of the solution, colorless block-shaped single crystals formed within a few hours. SCXRD analysis confirmed the formation of a dinuclear bis-silylene Ag complex [LSi{Ag(Mes)}–(NDipp)–{Ag(Mes)}SiL] (3) (Fig. 2). The previously non-symmetrical silaiminyl-silylene rearranged to a symmetrical bis-silylene *via* Si(i)–Si(iii) bond cleavage and Si(ii)–N bond formation. The narrow Si–N–Si angle of the *cis*-conformer of the newly formed bis-silylene ligand facilitated a semi-supported argentophilic interaction of 3.1235(3) Å between the two Ag(i) atoms, which is compatible with the values reported in the literature (2.9–3.4 Å).^30,31^ Both Ag atoms exhibit similar, nearly linear coordination environments, with Si1–Ag–C1 and Si2–Ag2–C10 angles being 164.11(5)° and 165.52(5)°, respectively. The Ag–silylene distances found in complex 3 are 2.3993(5) (Ag1–Si1) and 2.4191(5) Å (Ag2–Si2), slightly longer than in complex 1 (2.3847(5) Å). At room temperature, the NMR spectrum of complex 3 exhibited multiple sets of signals, posing challenges for signal assignment. To gain deeper insight into the behavior in solution, variable-temperature (VT) NMR experiments were conducted from 298 K to 348 K (Fig. S7). At 348 K, both the *t*Bu and Dipp protons displayed only one set of signals, indicating the presence of a symmetrical species in solution, which is consistent with its molecular structure (Fig. S8). In the ^29^Si{^1^H} NMR spectrum, a broad doublet signal was detected at 19.7 ppm with a ^1^*J*_29Si–107/109Ag_ value of 308 Hz. Compared to the silylene signal in compound 1 (48.5 ppm) and in the free silaiminyl-silylene (31.8 ppm), the signal found here is shifted to lower frequencies upon rearrangement to the bis-silylene and coordination to two Ag fragments.

**Fig. 3:**
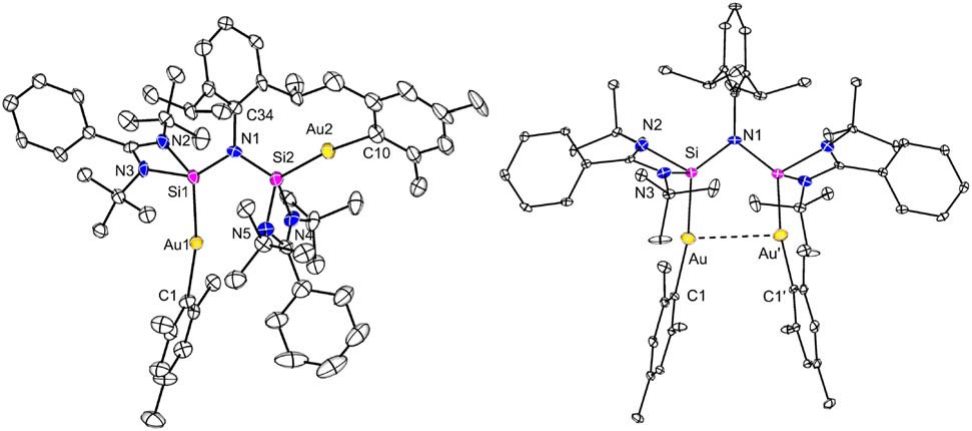
Molecular structures of complexes 4 (left) and 4′ (right) in the solid state. All hydrogen atoms and non-coordinating solvents are omitted for clarity. Selected bond distances [Å] and angles [°] in 4: Au1–Si1 2.318(2), Au2–Si2 2.307(2), Au1–C1 2.096(8), Au1–C10 2.072(8), Si1–N1 1.778(7), Si2–N1 1.759(7), N1–C34 1.478(10); Si1–Au1–C1 169.3(2), Si2–Au2–C10 171.9(3), Si1–N1–Si2 120.1(4); in 4′: Au⋯Au′ 3.4545(4), Au–Si 2.3072(10), Au–C1 2.066(4), Si1–N1 1.764(3); Si–Au–C1 170.56(11), Si–N1–Si′ 105.0(2).

Similarly, [AuMes]_5_ was reacted with the mononuclear gold complex 2 under the same conditions as for the Ag complex (Scheme 3). Light-orange single crystals of the binuclear Au complex 4 were obtained from a concentrated C_6_D_6_ solution at room temperature in 58% yield. X-ray diffraction analysis indicated the rearrangement of the ligand moiety and the formation of a bis-silylene-supported dinuclear Au complex [LSi{Au(Mes)}–(NDipp)–{Au(Mes)}SiL] (4) (Fig. 3).

Interestingly, unlike dinuclear Ag complex 3, the two silylene moieties in 4 did not arrange in a *cis*-configuration. Instead, the two silylene subunits are pointing to different directions in a *trans*-arrangement, which is likely due to the minimization of the steric repulsion and packing effects in the solid-state (see below for quantum chemical calculations).

Consequently, complex 4 displays no aurophilic interaction, which is at first glance counterintuitive. Both Si atoms adopt distorted tetrahedral geometries and the Au atoms are linearly coordinated by the silylene and the mesityl groups with Si1–Au1–C1 and Si2–Au2–C8 angles being 169.3(2)° and 171.9(3)°, respectively. The Au–Si bond lengths found in complex 4 (Au1–Si1 2.318(2) and Au2–Si2 2.307(2) Å) are similar to those in the silaimilyl-silylene Au complex 2 (2.3006(8) Å). The NMR spectra (^1^H, ^13^C{^1^H}, ^29^Si{^1^H}) were recorded at 343 K. The ^1^H NMR spectrum reveals a singlet resonance for the *t*Bu group, and a broad singlet signal for the methyl protons from the Dipp substituent with a relative intensity of 3 : 1, revealing a highly symmetric species in solution (Fig. S12). In the ^29^Si{^1^H} NMR spectrum, a singlet signal was detected at 66.9 ppm, at higher frequencies compared to the dinuclear Ag compound 3. Interestingly, when the binuclear Au complex 4 was treated with ITMe (1,3,4,5-tetramethylimidazol-2-ylidene) in C_6_D_6_, the silaiminyl-silylene [LSi–Si(NDipp)L] and [(ITMe)Au(Mes)] (6) were formed. This clearly demonstrates the reversibility of the Lewis acid-induced rearrangement from [LSi–Si(NDipp)L] to the bis-silylene [LSi–(NDipp)–SiL]. Compound 6 was characterized by single-crystal XRD analysis and NMR spectroscopy (see the SI). In contrast, the reaction between the binuclear Ag complex 3 and ITMe yielded an unidentifiable mixture.

**Scheme 4:**
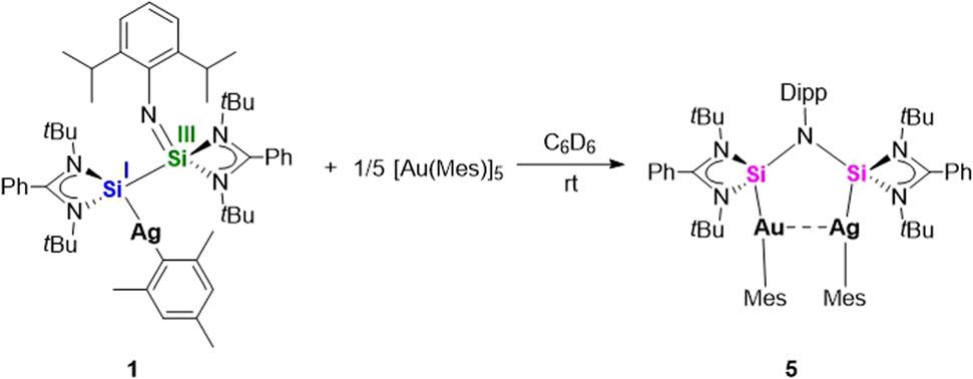
Synthesis of the heterobimetallic bis-silylene complex [LSi{Ag(Mes)}–(NDipp)–{Au(Mes)}SiL] (5).

In the continuity of our study, we aimed to generate a mixed-metal/bimetallic bis-silylene species. While a great number of dicarbene-bridged bimetallic complexes have been reported,^32–34^ general methods for such bimetallic bis-silylene complexes have remained elusive. Initially, the synthesis of bimetallic AgCu and AuCu complexes was attempted by reacting the mono-Ag complex 1 or the mono-Au complex 2 with [CuMes]_5_. In the case of the Ag system 1, the reaction predominantly yielded the bis-silylene Cu complex [{(LSi)_2_(NDipp)}(CuMes)]^19^ as the major product. In contrast, the reaction involving the Au complex 2 resulted in a mixture of [{(LSi)_2_(NDipp)}(CuMes)] and the Au_2_ bis-silylene complex [LSi{Au(Mes)}–(NDipp)–{Au(Mes)}SiL] (4), as confirmed by NMR spectroscopy and single-crystal X-ray diffraction analysis. Interestingly, during one attempt, small amounts of the bis-Au complex crystallized in a *cis*-configuration (4′, Fig. 3) with an Au⋯Au separation of 3.4545(4) Å, which is structurally analogous to the bis-Ag complex 3. The crystallization of both the *trans*- and *cis*-configuration is consistent with their comparable energies (*vide infra*) and the broadened ^1^H resonances. Reaction between the mononuclear Ag complex 1 with [AuMes]_5_ or a similar reaction of the Au-containing compound 2 with [AgMes]_4_ successfully yielded the mixed bis-silylene Ag/Au product [LSi{Ag(Mes)}–(NDipp)–{Au(Mes)}SiL] (5) upon ligand rearrangement (Scheme 4). Both routes could afford complex 5 in similar yields.

**Fig. 4:**
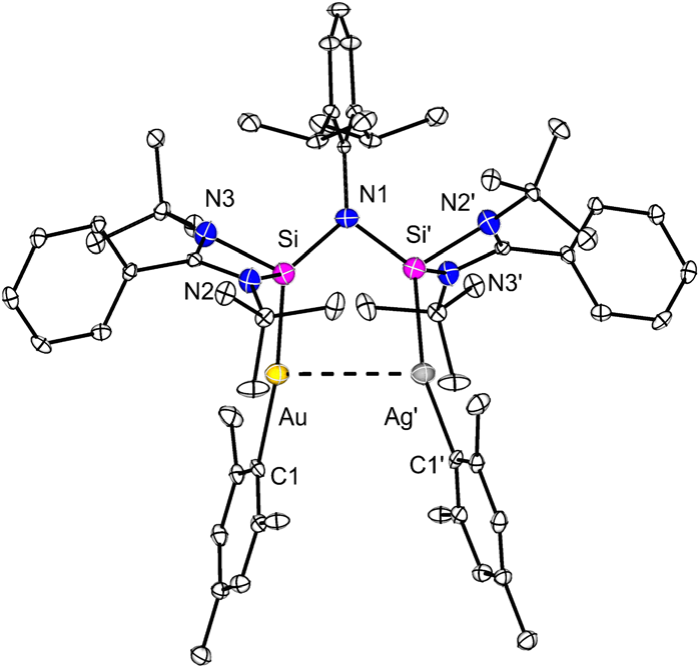
Molecular structures of complex 5 in the solid state. All hydrogen atoms and non-coordinating solvents are omitted for clarity. Selected bond distances [Å] and angles [°] in 5: Si–Ag′ 2.406(9), Si–Au 2.322(5), Au–C1 2.156(6), Ag′–C1′ 2.099(10); Si–Au–C1 172.0(2), Si′–Ag′–C1′ 165.0(4).

Yellow single crystals of compound 5 could be obtained after storage of the concentrated C_6_D_6_ solution at room temperature (Fig. 4). Notably, only half of the molecule is present in the asymmetric unit. Both Ag and Au metal positions were disordered, and this was modelled with 1 : 1 occupancy. Apart from the disorder of the two metals, the structural details are similar to those of the binuclear Ag complex 3 (Fig. 2). To further confirm that the isolated crystals only contain the mixed Ag/Au species 5 and do not consist of a mixture of the dinuclear Ag complex 3 and the dinuclear Au complex 4, ^1^H NMR spectroscopy was carried out (see SI, Fig. S15). Similar to compounds 3 and 4, only when rising to elevated temperature (343 K), meaningful but broad peaks can be detected. Peaks showing the asymmetric structure cannot be resolved. However, overlaying the ^1^H NMR spectrum of complexes 3, 4 and 5 clearly shows that the identities of all three compounds are different (see SI, Fig. S16). This can be seen, for example, from the different chemical shifts of the Dipp-CH protons (3.87 ppm for 3; 3.95 ppm for 4; 3.91 ppm for 5). The bis-silylene ligand in complex 5 is arranged in a *cis*-fashion, facilitating the metallophilic interaction between Ag and Au. However, upon keeping the solution of complex 5 at 343 K for a longer time, decomposition occurred, precluding the recording of the ^13^C{^1^H} and ^29^Si{^1^H} NMR spectra.

## Quantum chemical calculations

To investigate the bonding in the bimetallic complexes in more detail, quantum chemical investigations were carried out using the RI-DFT method (resolution of identity, density functional theory),^35–37^ with the addition of the D3 Grimme^38^ dispersion correction and def2-SV(P) basis sets for all atoms, as provided by the program package TURBOMOLE.^39,40^ Molecular orbital calculations (see SI, Fig. S34–S38) indicate that the HOMOs of the mononuclear complexes 1 and 2 feature significant contributions from the π system of the Si=N-Dipp moiety, while the LUMOs are mainly localized at the amidinate π* system. For the binuclear complexes 3 and 5, the HOMOs are localized on the Ag–C bonds, and the LUMOs are again centered at the amidinate π* system. In the case of the Au_2_ complex 4, the HOMO exhibits significant amidinate π character, and the LUMO is dominated by mesityl π* character. In particular, we were interested in why a *cis-*coordination at the bis-silylene ligand was realized in the case of the Ag_2_ and AuAg compounds 3 and 5, while a *trans*- and a *cis-*coordination were observed in the case of Au_2_ compounds 4 and 4′. For this purpose, both the *cis-* and *trans*-isomers were calculated for each of the three combinations, and their energies were compared. It was found that in all cases, the *cis*-configurated molecules are only slightly more stable than the *trans* ones (3: −5.5; 4′: −3.7; 5: −9.4 kJ mol^−1^). Thus, whether the *cis* or *trans* isomer was isolated could be a result of crystallization kinetics and not thermodynamically determined. The metal–metal distances of 3, 4′ and 5 are in very good agreement with the experimental data although the potential functions were found to be rather flat (3: 3.124 (exp.), 3.169 (theory); 4′: 3.455 (exp.), 3.281 (theory); 5: 3.371 (exp.), 3.333 Å (theory)). In expectation of a smaller cation radius of Au^+^ compared to Ag^+^, the gold–carbon and gold–silicon distances in these compounds are each about 4 pm shorter than for the corresponding Ag bonds. The torsion angles M–Si–Si–M′ (M, M′ = Ag, Au) in 3, 4′ and 5 of 40.1, 43.0 and 43.1°, respectively, indicate only a limited sterically demanding influence of the ligands in 4′ and 5.

Due to the weak metal–metal interaction, we performed an AIM analysis according to Bader's method in order to interpret the corresponding bond critical points in 3, 4′, and 5, respectively. The values for the electronic density at these bond critical points (*ρ* less than 0.02 a.u.) confirm these rather weak interactions. Recently, Frontera *et al.* worked intensively on a protocol to evaluate the energetic situation in argentophilic and aurophilic systems by means of AIM results.^41^ In their work, a methodology was derived to determine the binding energy of argentophilic and aurophilic bonds using the electron density at the bond critical points as a direct measure of the (covalent) shared electronic charge. Based on this research, for 3 and 4′, bond energies of only 19.5 and 15.4 kJ mol^−1^ were calculated.

Furthermore, local stretching force constants^42^ assessing the bond strength were determined by analyzing the Hessian matrices by means of the plugin LModeA-nano of the visualization program pymol.^43^ The metal–metal force constants of the Ag–Ag, Au–Au and Ag–Au bonds in the molecules under discussion were calculated to be 0.077 (3), 0.144 (4′) and 0.091 mdyn Å^−1^ (5), confirming weak interactions with respect to the strong single metal–metal bonds in the diatomics Ag_2_, Au_2_ and AgAu (exp. 1.17, 2.11 and 1.63 mdyn Å^−1^).^44^ To sum up, we assume that the bonding forces between the metal atoms are rather weak and lead to either *cis*- or *trans*-isomers, depending on their packing in the solid-state.

## Conclusion

In conclusion, our investigation into the reactivity and rearrangement of the silaiminyl-silylene with suitable silver and gold precursors in different stochiometric ratios has unveiled intriguing insights into its dynamic behavior and capability to act as a versatile silylene ligand. The isolation of the mono-silylene complexes 1 and 2 gave important evidence as intermediates of our previously proposed mechanism for the formation of the trigonal planar Cu bis-silylene complex. Moreover, these mononuclear complexes serve as starting materials to further access the bis-silylene coordinated homo-dinuclear complexes 3 and 4 and the hetero-dinuclear complex 5. In particular, the successful isolation of complex 5 underscores the unique ability of the silaiminyl-silylene to selectively coordinate two different coinage metals, forming a bimetallic bis-silylene complex, a feat unachievable with conventional bis-silylene ligands. Metallophilic interactions in the bimetallic compounds are rather weak in the range of packing effects.

## Author contributions

XS synthesized and analyzed all compounds with support from RY and DJ. FK synthesized the coinage metal precursors. RK performed and analyzed quantum chemical calculations. PR proposed the idea, supervised the work, and interpreted the results. All authors contributed to the manuscript.

## Conflicts of interest

There are no conflicts to declare.

## Supplementary Material

SC-016-D5SC04287A-s001

SC-016-D5SC04287A-s002

## Data Availability

All synthetic protocols, spectroscopic data, SI figures and tables, and detailed crystallographic information can be found in the SI. In detail, these include: synthesis and characterization, NMR and IR spectra, data or X-ray crystallographic studies, quantum chemical calculations. Crystallographic data are available *via* the Cambridge Crystallographic Data Centre (CCDC): 2393511–2393515 and 2448315–2448316. Data for this paper, including NMR and IR spectra as well as elemental analysis are available at radar4chem [https://radar.products.fiz-karlsruhe.de/] at https://doi.org/10.22000/8pmhqrnnewhqj0us. Synthesis and characterization, NMR, IR and UV-vis spectra, XRD data and calculations. See DOI: https://doi.org/10.1039/d5sc04287a.
