# Supplementary material for: On-demand switching from mono-silylene to bis-silylene to access mono-, di- and mixed coinage metal complexes
Source: Chem Sci. 2025 Aug 15;16(35):16057–62. doi: 10.1039/d5sc04287a (PMC12356211; doi:10.1039/d5sc04287a)
Supplement: SC-016-D5SC04287A-s001 [file SC-016-D5SC04287A-s001.pdf]

Supplementary Information (SI) for Chemical Science.
This journal is © The Royal Society of Chemistry 2025

On demand switching from mono-silylene to bis-silylene to access mono-, di- and mixed coinage metal complexes

Xiaofei Sun,*[a] Da Jin,[a] Ravi Yadav,[a,b] Frederic Kraetschmer,[a] Ralf Köppe,[a] Peter W. Roesky*[a]

[a] *Institute of Inorganic Chemistry (AOC), Karlsruhe Institute of Technology (KIT), Kaiserstr. 12, 76131, Germany.*

[b] School of Chemistry, Indian Institute of Science Education and Research Thiruvananthapuram, Thiruvananthapuram-695551, Kerala (India).

[c] Institute of Nanotechnology (INT), Karlsruhe Institute of Technology (KIT), Kaiserstr. 12, 76131, Germany.

Table of Contents

I. Synthesis and characterization S2

I.1 General procedures S2

I.2 Synthesis of [LSi{Ag(Mes)}–Si(NDipp)L] (**1**) S3

I.3 Synthesis of [LSi{Au(Mes)}–Si(NDipp)L] (**2**) S4

I.4 Synthesis of [LSi{Ag(Mes)}–(NDipp)–{Ag(Mes)}SiL] (**3**) S5

I.5 Synthesis of [LSi{Au(Mes)}–(NDipp)–{Au(Mes)}SiL] (**4**) S6

I.6 Synthesis of [LSi{Ag(Mes)}–(NDipp)–{Au(Mes)}SiL] (**5**) S7

I.7 Reaction between complex **4** and ITMe and formation of [(ITMe)Au(Mes)] (**6**) S8

II. NMR spectra S9

III. IR spectra S18

IV. X-ray crystallography S21

IV.1 General methods S21

IV.2 Summary of crystal data S22

IV.2 Crystal structures S24

V. Calculations S31

VI. UV-vis spectra S37

VII. References S40

I. Synthesis and characterization

I.1 General procedures

All air- and moisture-sensitive manipulations were performed under dry N_2 or Ar atmosphere using standard Schlenk techniques or in an argon-filled MBraun glovebox, unless otherwise stated. *n*-pentane and toluene were dried using an MBraun solvent purification system (SPS-800) and degassed. *n*-hexane was distilled under nitrogen from potassium benzophenone ketyl. C_6D_6 was dried over Na-K alloy and degassed by freeze-pump-thaw cycles. [LSi−Si(NDipp)L] (L = PhC(N*t*Bu)$_2$),[1] [AgMes]$_4$ (Mes = mesityl),[2] and [AuMes]$_5$[3] were prepared according to the literature procedures. All other chemicals were obtained from commercial sources and used without further purification.

Elemental analyses were carried out with an Elementar vario MICRO cube.

NMR spectra were recorded on Bruker spectrometers (Avance Neo 300 MHz, Avance Neo 400 MHz or Avance III 400 MHz). Chemical shifts are referenced internally using signals of the residual protio solvent (^{1}H) or the solvent (^{13}C{^{1}H}) and are reported relative to tetramethylsilane (^{1}H, ^{13}C{^{1}H}), or externally relative to tetramethylsilane (^{29}Si). All NMR spectra were measured at 298 K, unless otherwise specified. The multiplicity of the signals is indicated as s = singlet, d = doublet, dd = doublet of doublets, t = triplet, q = quartet, m = multiplet and br = broad. Assignments were determined based on unambiguous chemical shifts, coupling patterns and ^{13}C-DEPT experiments or 2D correlations (^{1}H-^{1}H COSY, ^{1}H-^{13}C HMQC and ^{1}H-^{13}C HMBC).

Infrared (IR) spectra were recorded in the region 4000–400 cm^{-1} on a Bruker Tensor 37 FTIR spectrometer equipped with a room temperature DLaTGS detector, a diamond attenuated total reflection (ATR) unit and a nitrogen-flushed chamber. In terms of their intensity, the signals were classified into different categories (vs = very strong, s = strong, m = medium, w = weak, and sh = shoulder).

I.2 Synthesis of [LSi{Ag(Mes)}–Si(NDipp)L] (1)

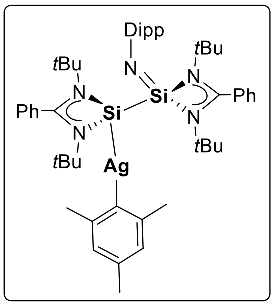

To a mixture of [LSi–Si(NDipp)L] (60.0 mg, 0.086 mmol) and $[AgMes]_4$ (19.6 mg, 0.022 mmol) was condensed *ca*. 5 mL of toluene at -88 °C. The mixture was allowed to warm up to room temperature and turned from light yellow to orange-red immediately. After keeping the mixture at room temperature for 5 min, the solution was concentrated to *ca*. 1 mL and kept at room temperature for crystallization. After several hours, yellow plates of complex **1** were formed, the solution was carefully removed and the isolated crystals were washed with small amounts of toluene and dried for 30 min under vacuum.

Crystalline yield: 40.5 mg (51%)

mp 150 °C (dec.).

^{1}H NMR (400.3 MHz, C_6D_6): *δ* (ppm) = 7.32-7.30 (m, 3H, CH_{Ar}), 7.21 (br, 2H, CH_{Ar}), 7.01-6.91 (m, 10H, CH_{Ar}), 4.46 (hept, $^1J(^1H-^1H)$ = 6.9 Hz, 2H, C*H* Dipp), 3.07 (s, 6H, *o*-CH_3), 2.44 (s, 6H, *p*-CH_3), 1.57 (d, $^1J(^1H-^1H)$ = 6.9 Hz, 12H, CH_3 Dipp), 1.22, 1.23 (two s, 36H, C(CH_3)$_3$).

$^{13}C\{^1H\}$ NMR (100.67 MHz, C_6D_6): *δ* (ppm) = 175.4 (C_q), 164.1 (C_q), 146.5 (C_q), 146.0 (C_q), 140.0 (C_q), 133.3 (C_q), 132.9 (C_q), 131.1 (C_q), 130.8, 130.7, 129.3 (C_q), 129.0, 128.8, 128.7, 128.6, 128.1, 127.7, 127.4, 125.1, 125.0, 122.7, 116.3, 54.6 (*C*(CH$_3$)$_3$), 54.1 (*C*(CH$_3$)$_3$), 32.0 (C(*C*H$_3$)$_3$), 30.7 (*o*-*C*H$_3$), 28.2 (*C*H Dipp), 25.2 (*C*H$_3$ Dipp), 21.9 (*p*-*C*H$_3$).

^{29}Si NMR (79.52 MHz, C_6D_6): *δ* (ppm) = 48.5 (d, $^1J(^{29}Si-^{107}Ag)$ = 188 Hz, $^1J(^{29}Si-^{109}Ag)$ = 217 Hz), -72.4 (d, $^2J(^{29}Si-^{107/109}Ag)$ = 28 Hz).

Anal. Calcd. For $C_{51}H_{74}AgN_5Si_2$ (921.23 g/mol): C 66.49; H 8.10; N 7.60. Found: C 66.95; H 8.04; N 7.23.

IR (ATR): $\tilde{\nu}$ (cm^{-1}) = 487 (w), 614 (w), 634 (w), 706 (m), 741 (m), 765 (m), 792 (w), 841 (w), 925 (w), 1021 (m), 1056 (w), 1085 (w), 1116 (vw), 1140 (vw), 1200 (s), 1257 (w), 1271 (m), 1361 (s), 1396 (vs), 1447 (s), 1470 (s), 1587 (w), 1646 (vw), 2862 (m), 2928 (m), 2957 (s), 3030 (vw), 3061 (vw).

I.3 Synthesis of [LSi{Au(Mes)}–Si(NDipp)L] (2)

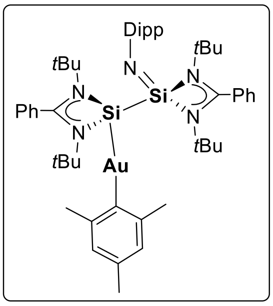

In a J. Young NMR tube containing [LSi–Si(NDipp)L] (50.0 mg, 0.072 mmol) and C_6D_6 was added $[AuMes]_5$ (22.8 mg, 0.014 mmol) in small portions. The solution changed from yellow to orange immediately. Subsequently, ^{1}H NMR spectrum was recorded and showed all starting materials were consumed and a set of new signals was formed. The solution was concentrated to *ca.* one third and kept at room temperature for crystallization. After several hours, yellow block-shaped crystals were formed. The solution was carefully decanted and the crystals were dried under vacuum for 30 min before storage in the glovebox.

Crystalline yield: 34 mg (47%).

mp 180 °C (dec.).

^{1}H NMR (400.3 MHz, C_6D_6): *δ* (ppm) = 7.36-7.28 (m, 5H, CH_{Ar}), 7.20-7.18 (m, 2H, CH_{Ar}), 7.07-7.02 (m, 2H, CH_{Ar}), 6.97-6.91 (m, 6H, CH_{Ar}), 4.51 (hept, $^1J(^1H\text{-}^1H)$ = 6.8 Hz, 2H, C*H* Dipp), 3.13 (s, 6H, *o*-CH_3), 2.43 (s, 3H, *p*-CH_3), 1.58 (d, $^1J(^1H\text{-}^1H)$ = 6.8 Hz, 12H, CH_3 Dipp), 1.27, 1.26 (two s, 36H, $C(CH_3)_3$).

^{13}C{^{1}H} NMR (100.67 MHz, C_6D_6): *δ* (ppm) = 145.9, 140.2, 135.5, 127.1, 122.8, 54.6 (*C*$(CH_3)_3$), 54.2 (*C*$(CH_3)_3$), 32.0 ($C(CH_3)_3$), 31.8 ($C(CH_3)_3$), 28.1 (*C*H Dipp), 27.4 (*o*-CH_3), 25.3 (CH_3 Dipp), 21.7 (*p*-CH_3).

Due to the very poor solubility of the complex in C_6D_6, despite long measurement time of the ^{13}C{^{1}H} NMR spectrum (number of scans = 21433), several aromatic proton signals could not be detected. In the ^{29}Si{^{1}H} NMR spectrum, no signals were detected.

The sample decomposes during the course of three days at room temperature in C_6D_6 and decomposes immediately in THF-d_8 or CD_2Cl_2.

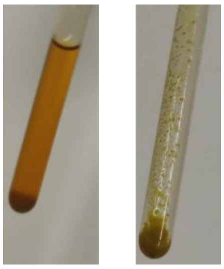

Figure S1. Left: Photograph of isolated crystals of complex **2** (20 mg) in C_6D_6, showing the bad solubility of the species. Right: After decantation of the solvent, the remaining solid are crystalline.

Anal. Calcd. For $C_{51}H_{74}AuN_5Si_2$ C_6D_6 (1079.44 g/mol): C 62.55; H 7.92; N 6.40. Found: C 62.94; H 7.43; N 6.20.

IR (ATR): $\tilde{\nu}$ (cm^{-1}) = 3057 (w), 2959 (s), 2932 (s), 2912 (s), 2871 (m), 1644 (w), 1599 (w), 1577 (w), 1515 (w), 1448 (s), 1392 (vs), 1363 (s), 1312 (m), 1249 (m), 1220 (m), 1189 (s), 1159 (m), 1093 (m), 1081 (m), 1023 (m), 929 (w), 876 (s), 860 (s), 839 (s), 792 (s), 757 (m), 733 (m), 706 (s), 630 (m), 538 (m), 485 (m), 434 (m).

I.4 Synthesis of [LSi{Ag(Mes)}–(NDipp)–{Ag(Mes)}SiL] (3)

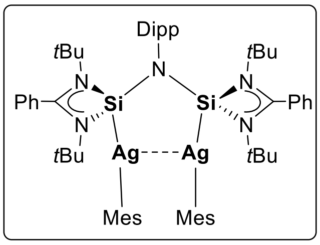

To a J. Young NMR tube containing a C_6D_6 solution of [LSi{Ag(Mes)}–Si(NDipp)L] (**1**) (45 mg, 0.049 mmol) was added $[AgMes]_4$ (11.1 mg, 0.012 mmol) and the resulting solution turned from orange-red to light orange. ^{1}H NMR spectroscopy confirmed the reaction took place already within 5 min. The solution was concentrated to *ca.* one third and was kept at room temperature for crystallization. After a few hours, block-shaped colorless crystals were formed. The solution was carefully decanted and the crystals were dried under vacuum for 30 min.

Crystalline yield: 25 mg (54%).

mp 195 °C (dec.).

^{1}H NMR (400.3 MHz, C_6D_6, 348 K): δ (ppm) = 7.47 (br s, 2H, CH_{Ar}), 7.14-7.07 (m, 9H, CH_{Ar}), 6.95 (br s, 4H, CH_{Ar}), 6.84 (br s, 2H, CH_{Ar}), 3.87 (hept, $^1J(^1H-^1H)$ = 6.8 Hz, 2H, C*H* Dipp), 2.84 (s, 12H, *o*-CH_3), 2.37, 2.34 (s, 6H, *p*-CH_3), 1.51 (br s, 12H, CH_3 Dipp), 1.32 (br s, 36H, C(CH_3)$_3$).

^{13}C{^{1}H} NMR (100.67 MHz, C_6D_6, 343 K): δ (ppm) = 171.6 (br, C_q, N*C*N), 146.8 (br, C_q), 146.0 (C_q), 133.5 (C_q), 132.6 (C_q), 130.5, 129.0 (br), 128.6, 128.4, 128.0, 127.7 (br), 125.4, 125.1, 55.7 (*C*(CH$_3$)$_3$), 33.3 (C(*C*H$_3$)$_3$), 30.8 (*o*-*C*H$_3$), 23.4 (br, *C*H Dipp + *C*H$_3$ Dipp), 21.5 (*p*-*C*H$_3$). Two C_q signals could not be detected.

^{29}Si NMR (79.52 MHz, C_6D_6, 343 K): δ (ppm) = 19.7 (br d, $^1J(^{29}Si-^{107/109}Ag) \approx 308$ Hz).

Anal. Calcd. For $C_{60}H_{85}Ag_2N_5Si_2$ (1148.28 g/mol): C 62.76; H 7.46; N 6.10. Found: C 62.51; H 6.71; N 6.14.

IR (ATR): $\tilde{\nu}$ (cm^{-1}) = 2959 (s), 2930 (m), 2910 (m), 2871 (m), 1445 (m), 1427 (m), 1390 (vs), 1363 (s), 1263 (w), 1230 (w), 1195 (s), 1163 (w), 1146 (w), 1099 (m), 1079 (w), 1025 (w), 927 (w), 901 (m), 878 (w), 843 (w), 823 (w), 788 (m), 761 (w), 745 (m), 731 (m), 704 (m), 616 (w), 530 (w), 493 (w), 438 (vw), 423 (w).

I.5 Synthesis of [LSi{Au(Mes)}–(NDipp)–{Au(Mes)}SiL] (4)

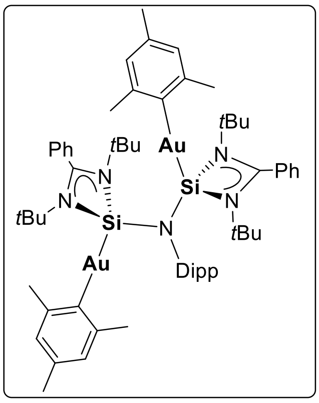

To a J. Young NMR tube containing a C_6D_6 solution of [LSi{Au(Mes)}–Si(NDipp)L] (**2**) (30 mg, 0.030 mmol) was added $[AuMes]_5$ (9.5 mg, 0.012 mmol) and the resulting solution turned from orange-red to light orange. ^{1}H NMR spectroscopy confirmed the reaction took place already within 5 min. The solution was concentrated to *ca.* one third and was kept at room temperature for crystallization. After a few hours, plate-shaped light orange crystals were formed. The solution was carefully decanted and the crystals were dried under vacuum for 30 min.

Crystalline yield: 23 mg (58%).

mp 215 °C (dec.).

^{1}**H NMR** (400.3 MHz, C_6D_6, 343 K): δ (ppm) = 7.56 (br s, 2H, CH_{Ar}), 7.23-7.21 (m, 3H, CH_{Ar}), 7.14-7.12 (m, 6H, CH_{Ar}), 6.94 (br s, 4H, CH_{Ar}), 6.85 (br s, 2H, CH_{Ar}), 3.95 (hept, $^{1}J(^{1}H-^{1}H)$ = 6.8 Hz, 2H, C*H* Dipp), 2.85 (s, 12H, *o*-CH_3), 2.37, 2.35 (s, 6H, *p*-CH_3), 1.61 (br s, 12H, CH_3 Dipp), 1.38 (br s, 36H, $C(CH_3)_3$).

^{13}**C{^{1}H} NMR** (100.67 MHz, C_6D_6, 343 K): δ (ppm) = 145.9 (C_q), 133.7 (C_q), 132.8 (C_q), 130.5, 129.2 (C_q), 128.4, 127.9, 127.5, 127.0, 125.4, 55.7 ($C(CH_3)_3$), 33.0 ($C(CH_3)_3$), 29.3 (*C*H Dipp), 28.3 (*C*H_3 Dipp), 27.4 (*o*-CH_3), 21.6 (*p*-CH_3). Three C_q and two CH_{Ar} signals could not be detected.

^{29}**Si{^{1}H} NMR** (79.52 MHz, C_6D_6, 343 K): (ppm) = 66.9.

Anal. Calcd. For $C_{60}H_{85}Au_2N_5Si_2$ C_6D_6 (1410.63 g/mol): C 56.20; H 6.93; N 4.96. Found: C 56.17; H 6.62; N 4.97.

IR (ATR): $\tilde{\nu}$ (cm^{-1}) = 2959 (s), 2910 (s), 2869 (s), 1515 (m), 1445 (s), 1392 (vs), 1363 (s), 1331 (m), 1318 (m), 1281 (m), 1253 (m), 1224 (m), 1193 (s), 1157 (m), 1093 (m), 1081 (m), 1021 (m), 929 (m), 874 (s), 860 (s), 843 (s), 792 (s), 757 (s), 739 (m), 706 (s), 630 (m), 600 (w), 536 (m), 495 (m), 444 (w).

I.6 Synthesis of [LSi{Ag(Mes)}–(NDipp)–{Au(Mes)}SiL] (5)

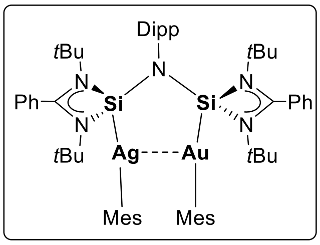

To a J. Young NMR tube containing a C_6D_6 solution of [LSi{Ag(Mes)}–Si(NDipp)L] (**1**) (35 mg, 0.038 mmol) was added $[AuMes]_5$ (12.0 mg, 0.008 mmol) and the solution was sonicated for 5 min at room temperature. Recording the ^{1}H NMR spectrum showed that all starting materials were consumed. The solution was concentrated and was kept at room temperature for crystallization. After a few hours, single crystals have formed. The solution was carefully decanted and the crystals were dried under vacuum for 30 min.

Crystalline yield: 23 mg (44%).

mp 160 °C (dec.).

^{1}H NMR (400.3 MHz, C_6D_6, 343 K): δ (ppm) = 7.51 (br s, 2H, CH_{Ar}), 7.21-7.10 (m, 9H, CH_{Ar}), 6.94 (br s, 4H, CH_{Ar}), 6.85 (br s, 2H, CH_{Ar}), 3.91 (hept, $^1J(^1H-^1H)$ = 6.8 Hz, 2H, C*H* Dipp), 2.84 (s, 12H, *o*-CH_3), 2.38 (s, 6H, *p*-CH_3), 2.33 (s, 6H, *p*-CH_3), 1.51 (br s, 12H, CH_3 Dipp), 1.34 (br s, 36H, $C(CH_3)_3$).

Anal. Calcd. For $C_{60}H_{85}AgAuN_5Si_2$ 0.5 C_6D_6 (1279.46 g/mol): C 59.14; H 7.17; N 5.47. Found: C 59.70; H 6.85; N 5.25.

Due to the very poor solubility of the complex in C_6D_6, despite long measurement time of the $^{13}C\{^1H\}$ NMR spectrum at high temperatures, no signals were detected. For the same reason no $^{29}Si\{^1H\}$ NMR spectrum was recorded. The sample decomposes in C_6D_6 at high temperature after several hours and decomposes immediately in THF-d_8 or CD_2Cl_2.

IR (ATR): $\tilde{\nu}$ (cm^{-1}) = 3057 (w), 2959 (vs), 2930 (s), 2869 (s), 2164 (w), 1644 (s), 1599 (m), 1578 (m), 1515 (w), 1443 (s), 1392 (vs), 1359 (s), 1314 (m), 1284 (m), 1255 (s), 1222 (s), 1195 (s), 1163 (m), 1140 (m), 1099 (m), 1077 (s), 1042 (s), 1021 (s), 1001 (s), 929 (m), 905 (m), 874 (s), 843 (m), 790 (s), 743 (s), 704 (s), 630 (m), 622 (m), 495 (w), 432 (w).

I.7 Reaction between complex 4 and ITMe and formation of [(ITMe)Au(Mes)] (6)

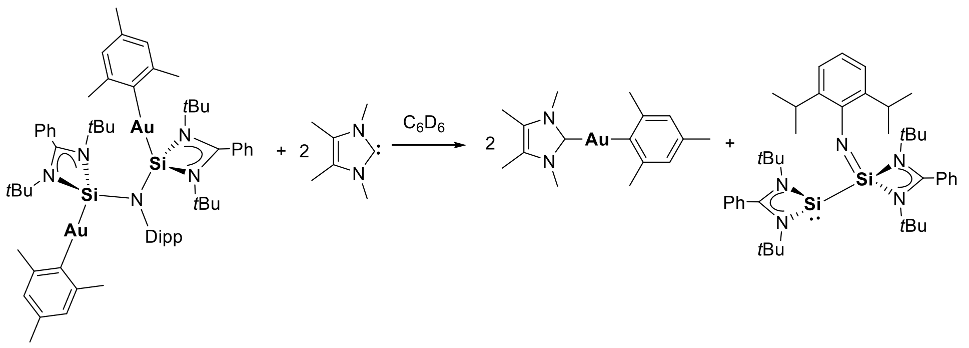

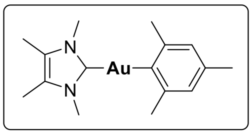

To a J. Young NMR tube containing a C_6D_6 solution of [LSi{Au(Mes)}–(NDipp)–{Au(Mes)}SiL] (**4**) (30 mg, 0.021 mmol) was added ITMe (6.0 mg, 0.047 mmol) and the solution was sonicated for 5 min at room temperature and kept at room temperature for 4 h. The solution was concentrated and kept at room temperature for crystallization. After a few days, single crystals have formed. The solution was carefully decanted and the crystals were dried under vacuum for 30 min.

Crystalline yield: 10 mg (54%).

^{1}H NMR (400.3 MHz, THF-d_8): δ (ppm) = 6.65 (s, 2H, C*H*, mesityl), 3.78 (s, 6H, N-CH_3, NHC), 2.47 (s, 6H, *o*-CH_3 mesityl), 2.15 (s, 6H, C-CH_3, NHC), 2.14 (s, 6H, *p*-CH_3 mesityl).

^{13}C{^{1}H} NMR (100.67 MHz, THF-d_8): δ (ppm) = 195.0 (N*C*N), 169.7 (C_{ipso} mesityl), 146.3 (*o*-C_q mesityl), 132.7 (*p*-C_q mesityl), 126.2 (*m*-*C*H, mesityl), 125.5 (C_q, NHC), 34.7 (N-*C*H$_3$, NHC), 27.4 (*o*-*C*H$_3$ mesityl), 21.5 (*p*-*C*H$_3$ mesityl), 8.8 (C-C*H*$_3$, NHC).

IR (ATR): $\tilde{\nu}$ (cm^{-1}) = 2965 (s), 2922 (vs), 2906 (vs), 2857 (m), 2771 (w), 1651 (m), 1581 (w), 1542 (w), 1446 (vs) , 1391 (vs), 1370 (m), 1360 (m), 1261 (w), 1229 (w), 1167 (w), 1102 (w), 1028 (w), 846 (s), 745 (w), 717 (w), 631 (w), 578 (w), 551 (w).

II. NMR spectra

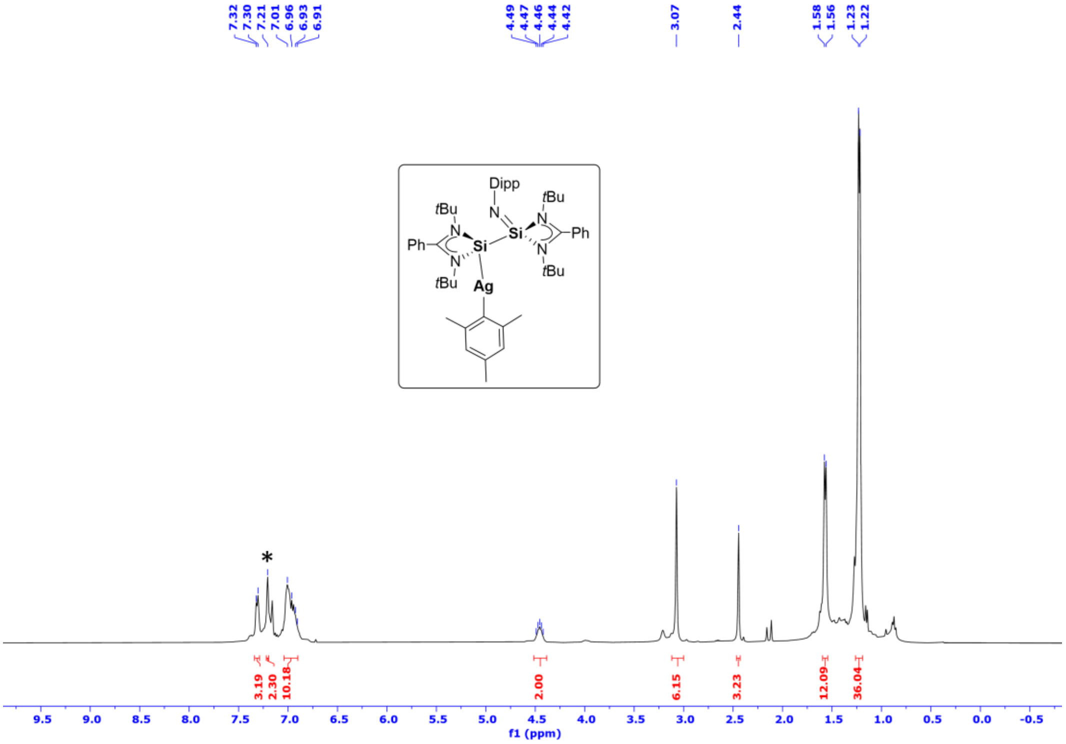

Figure S2. ^{1}H NMR spectrum of [LSi{Ag(Mes)}–Si(NDipp)L] (**1**) in C_6D_6. *, residual protio solvent signal.

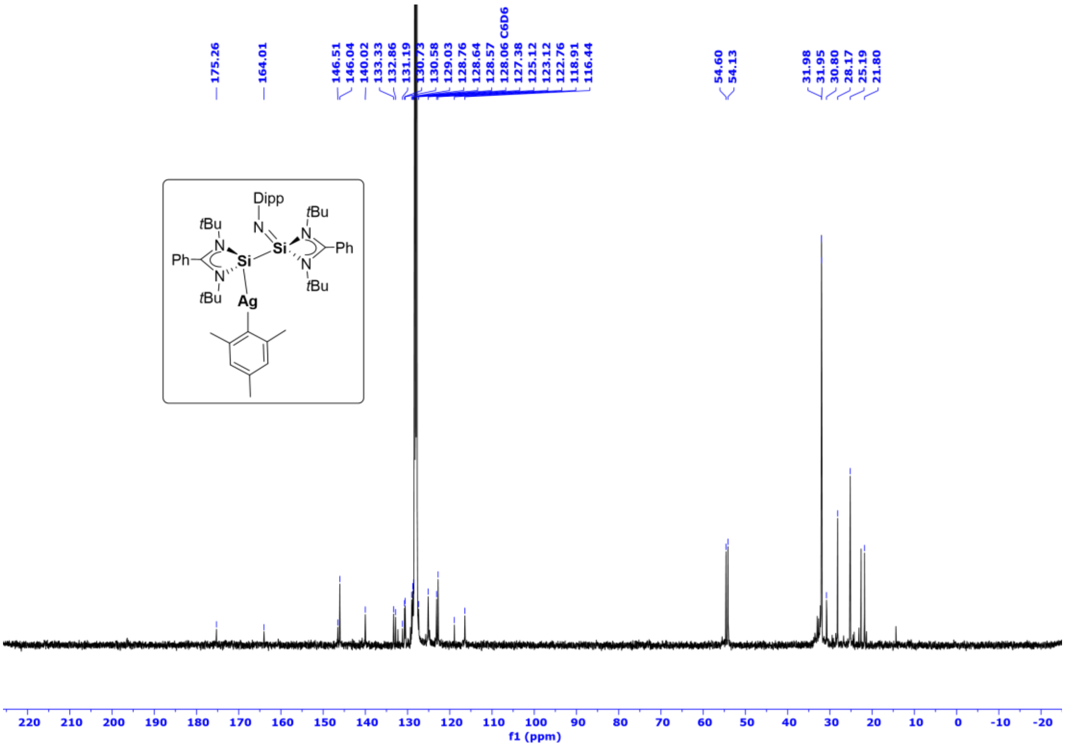

Figure S3. $^{13}C\{^{1}H\}$ NMR spectrum of [LSi{Ag(Mes)}–Si(NDipp)L] (**1**) in C_6D_6.

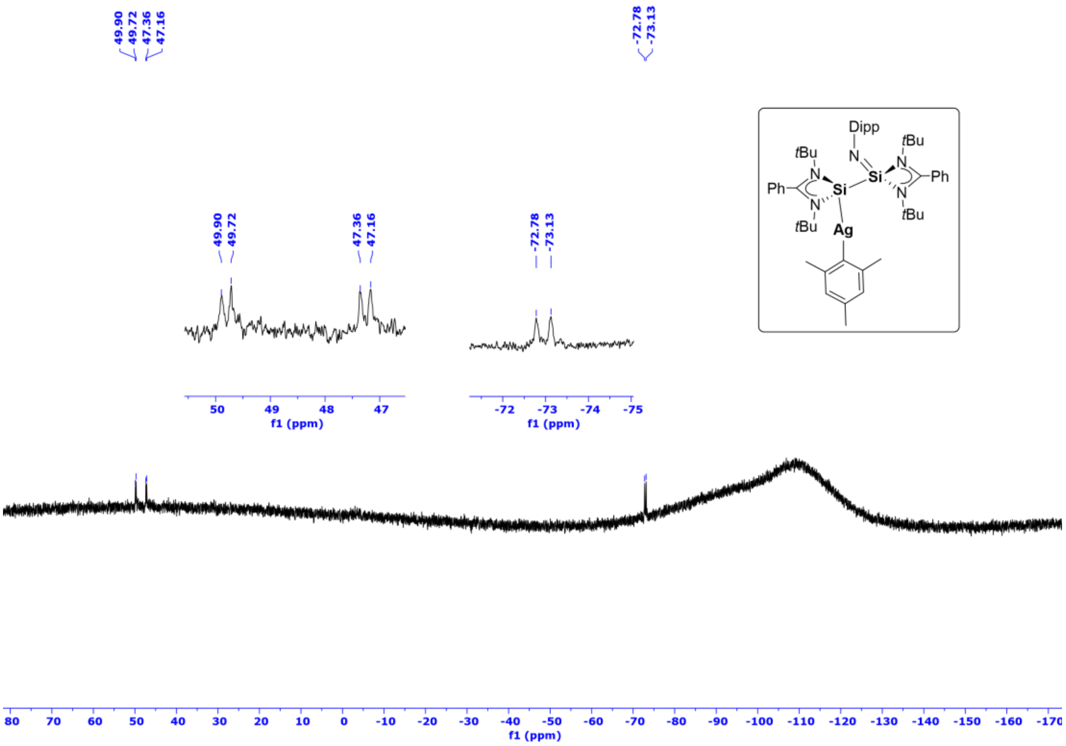

Figure S4. $^{29}Si\{^1H\}$ NMR spectrum of [LSi{Ag(Mes)}–Si(NDipp)L] (**1**) in C_6D_6.

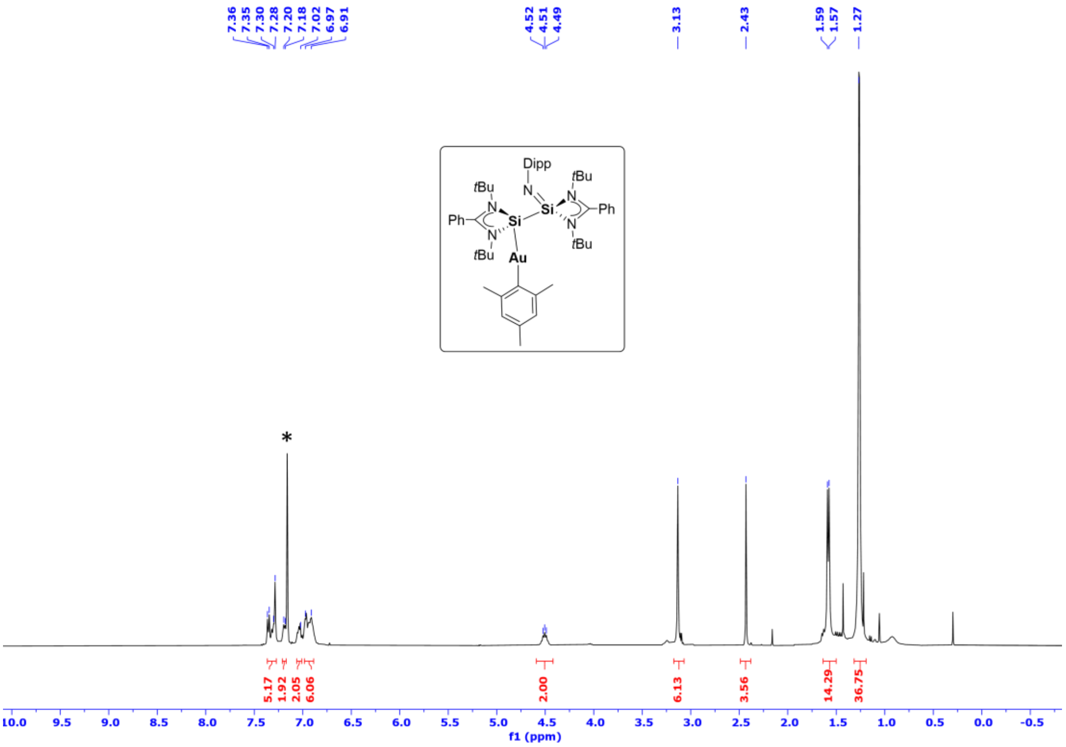

Figure S5. ^1H NMR spectrum of [LSi{Au(Mes)}–Si(NDipp)L] (**2**) in C_6D_6. *, residual protio solvent signal.

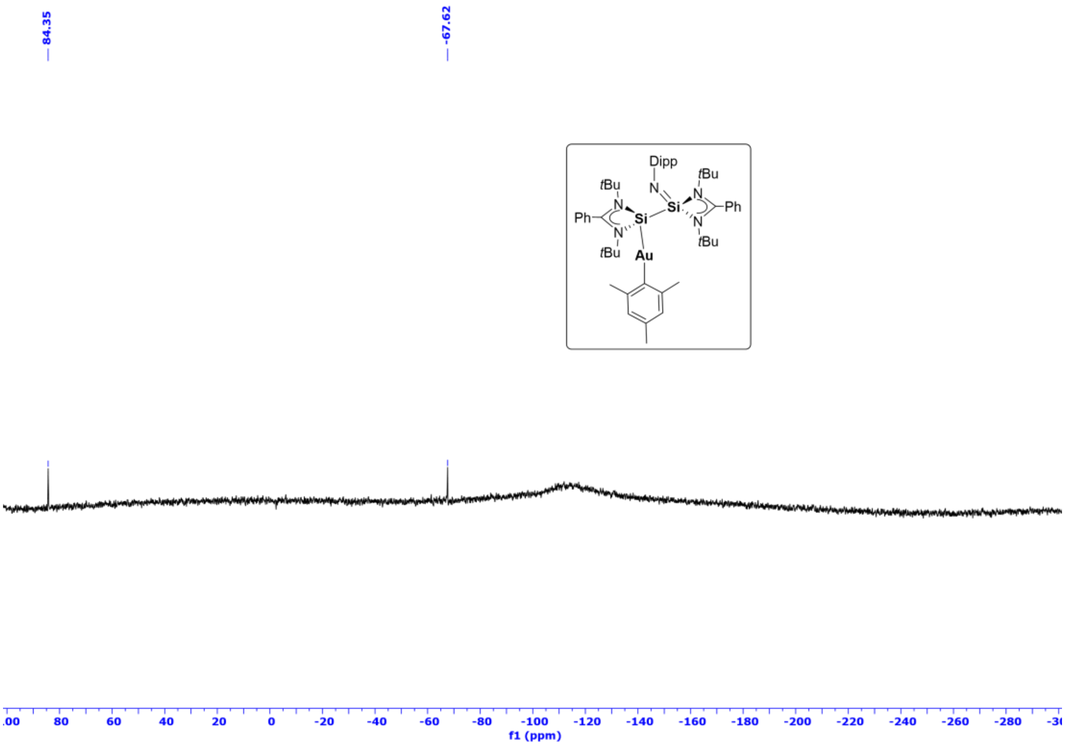

Figure S6 $^{29}Si\{^{1}H\}$ NMR spectrum of the NMR reaction between [LSi–Si(NDipp)L] and $[Au(Mes)]_5$ in C_6D_6 recorded after 2 h reaction time.

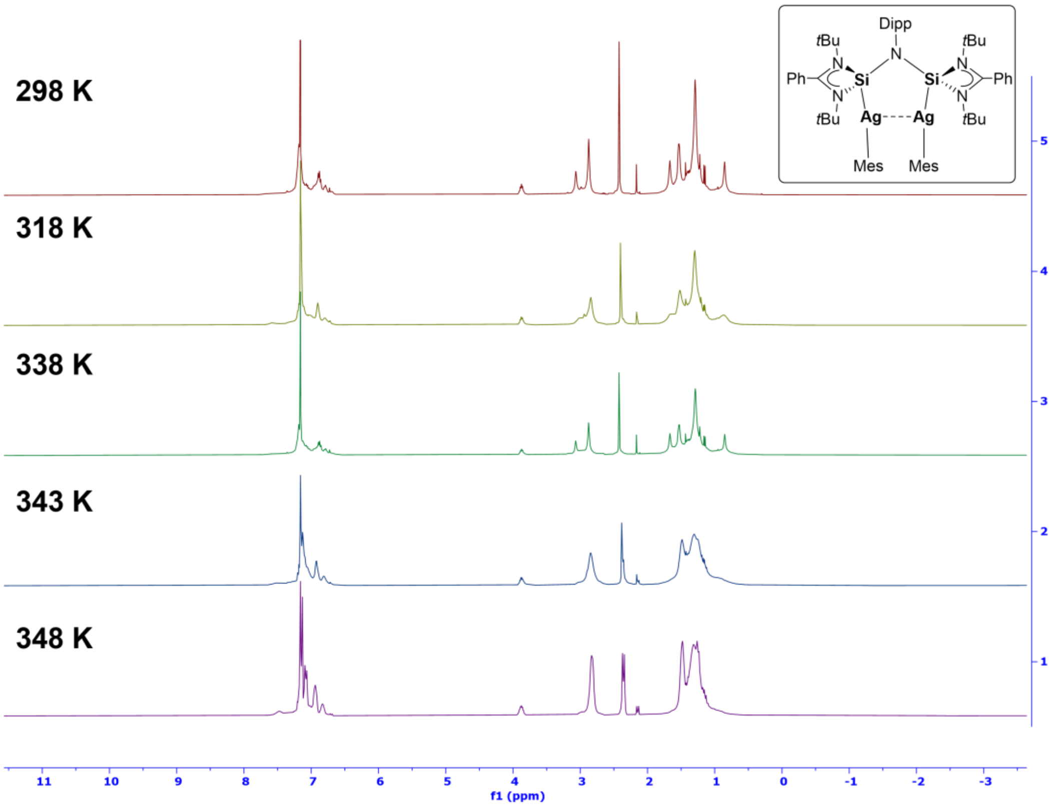

Figure S7. ^{1}H NMR spectrum of [LSi{Ag(Mes)}−(NDipp)−{Ag(Mes)}SiL] (**3**) in C_6D_6 measured from 298 K to 348 K.

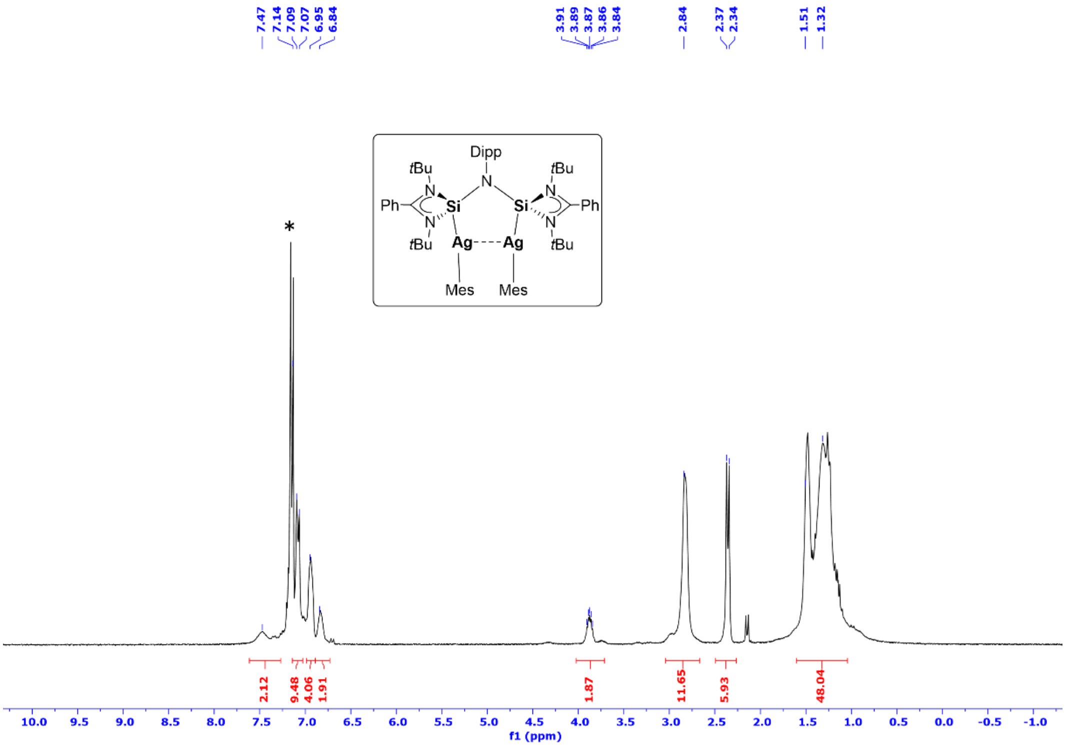

Figure S8. ^{1}H NMR spectrum of [LSi{Ag(Mes)}–(NDipp)–{Ag(Mes)}SiL] (**3**) in C_6D_6 measured at 348 K. *, residual protio solvent signal.

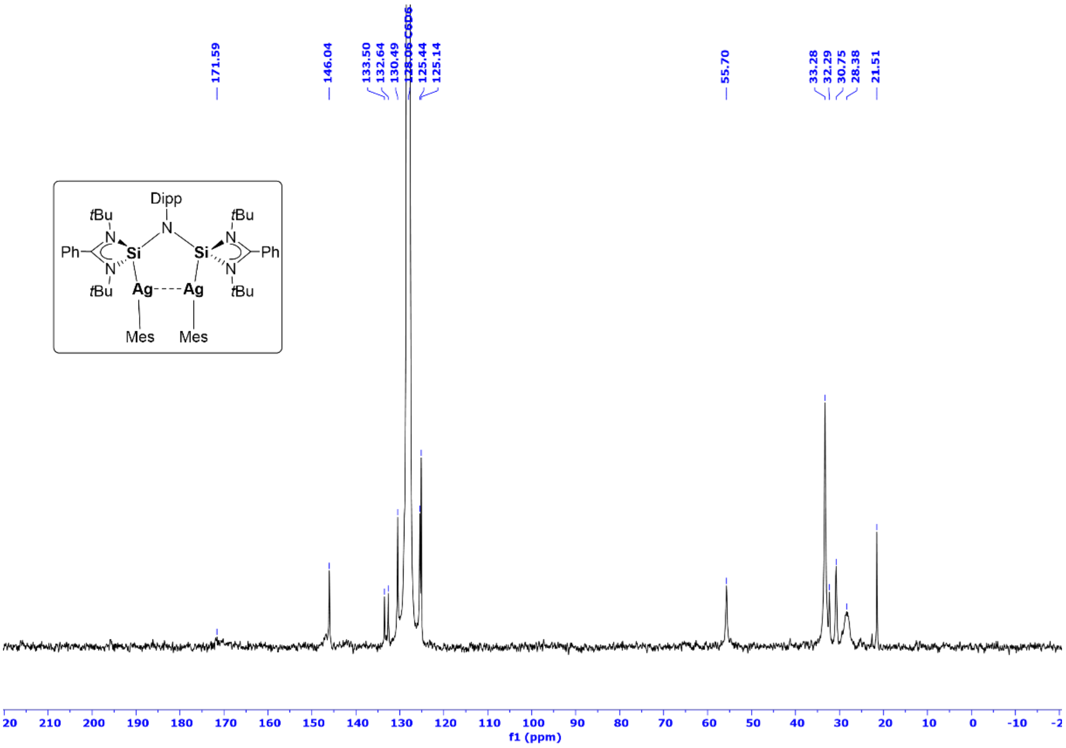

Figure S9. $^{13}C\{^{1}H\}$ NMR spectrum of [LSi{Ag(Mes)}–(NDipp)–{Ag(Mes)}SiL] (**3**) in C_6D_6 measured at 348 K.

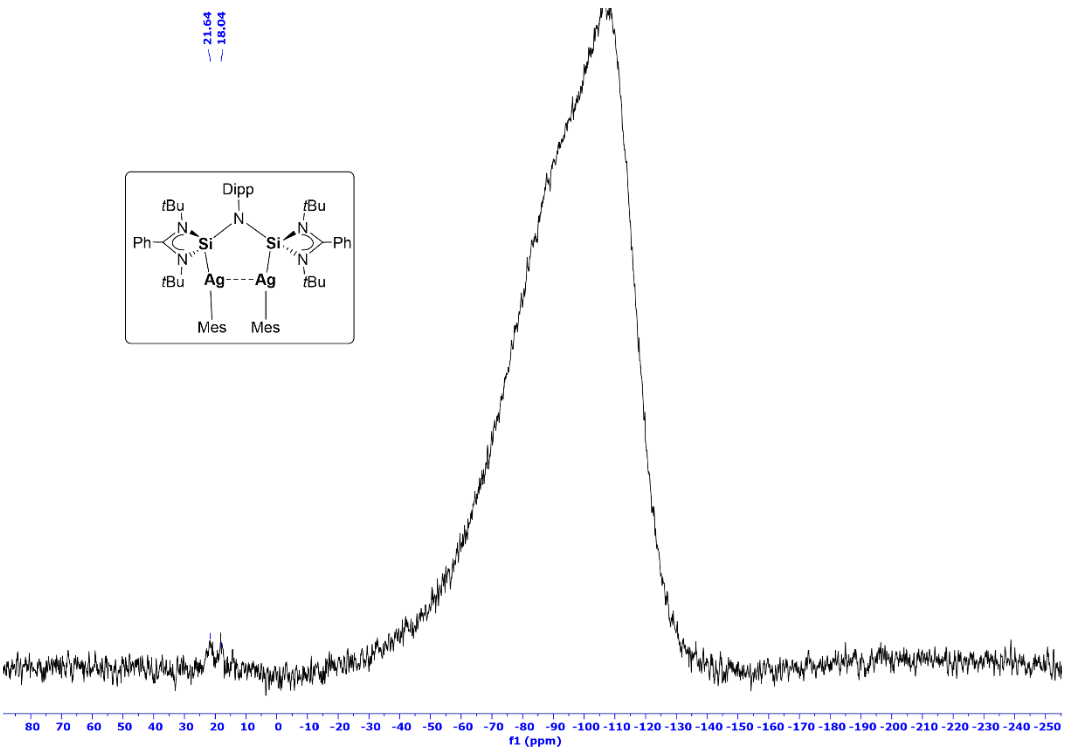

Figure S10. $^{29}Si\{^1H\}$ NMR spectrum of [LSi{Ag(Mes)}–(NDipp)–{Ag(Mes)}SiL] (**3**) in C_6D_6 measured at 343 K.

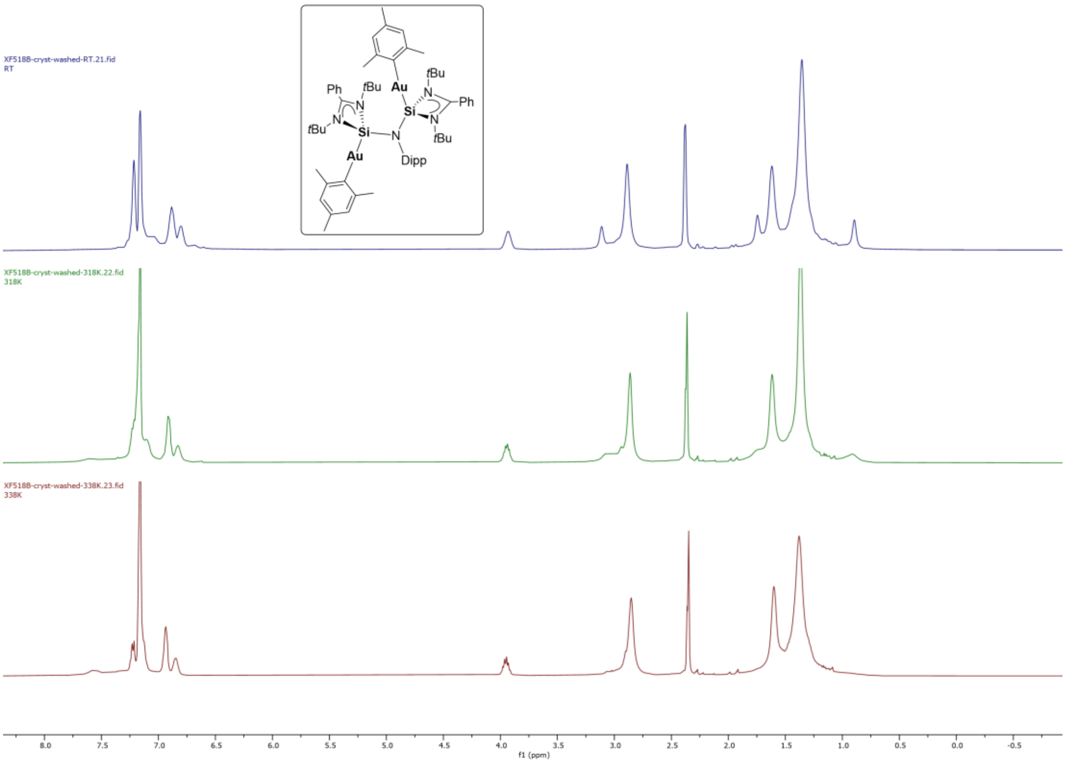

Figure S11. ^1H NMR spectrum of [LSi{Au(Mes)}–(NDipp)–{Au(Mes)}SiL] (**4**) in C_6D_6 measured at 298 K (top), 318 K (middle) and 338 K (bottom). *, residual protio solvent signal.

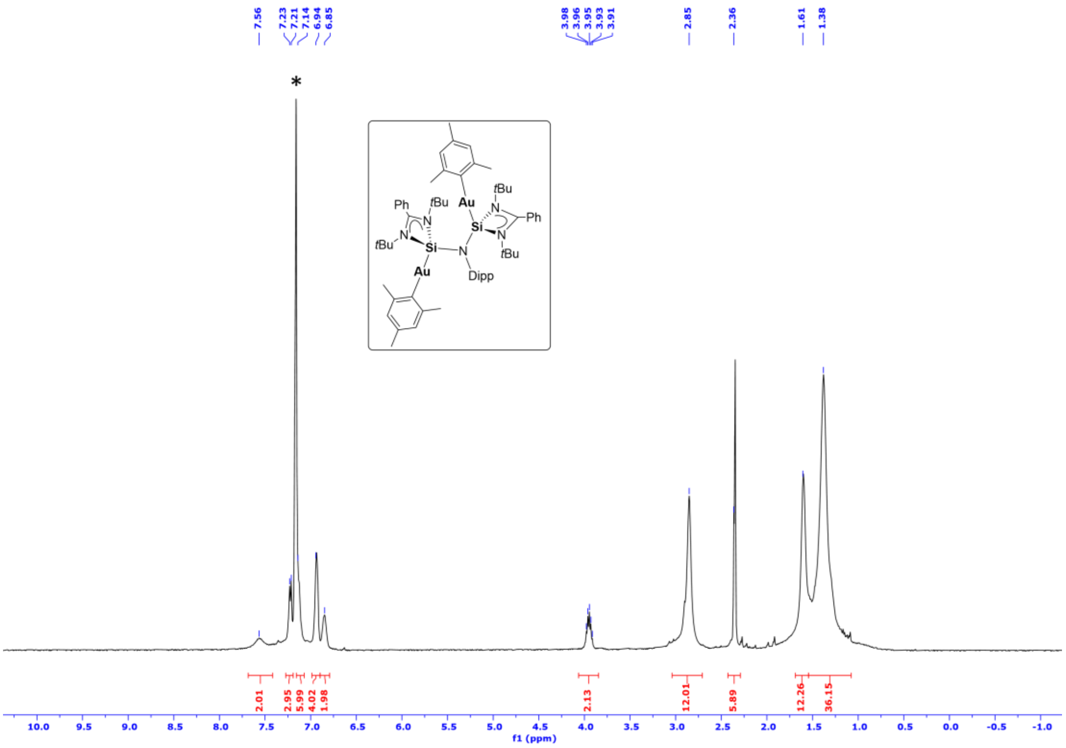

Figure S12. ^{1}H NMR spectrum of [LSi{Au(Mes)}−(NDipp)−{Au(Mes)}SiL] (**4**) in C_6D_6 measured at 343 K. *, residual protio solvent signal.

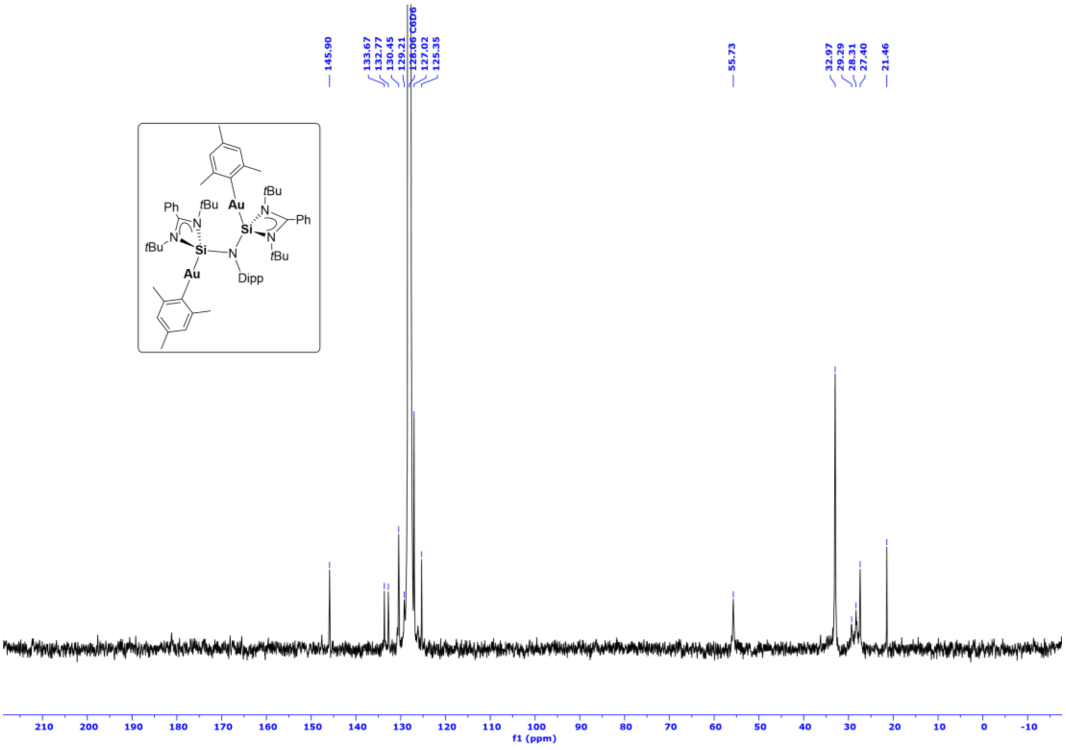

Figure S13. ^{13}C{^{1}H} NMR spectrum of [LSi{Au(Mes)}−(NDipp)−{Au(Mes)}SiL] (**4**) in C_6D_6 measured at 343 K.

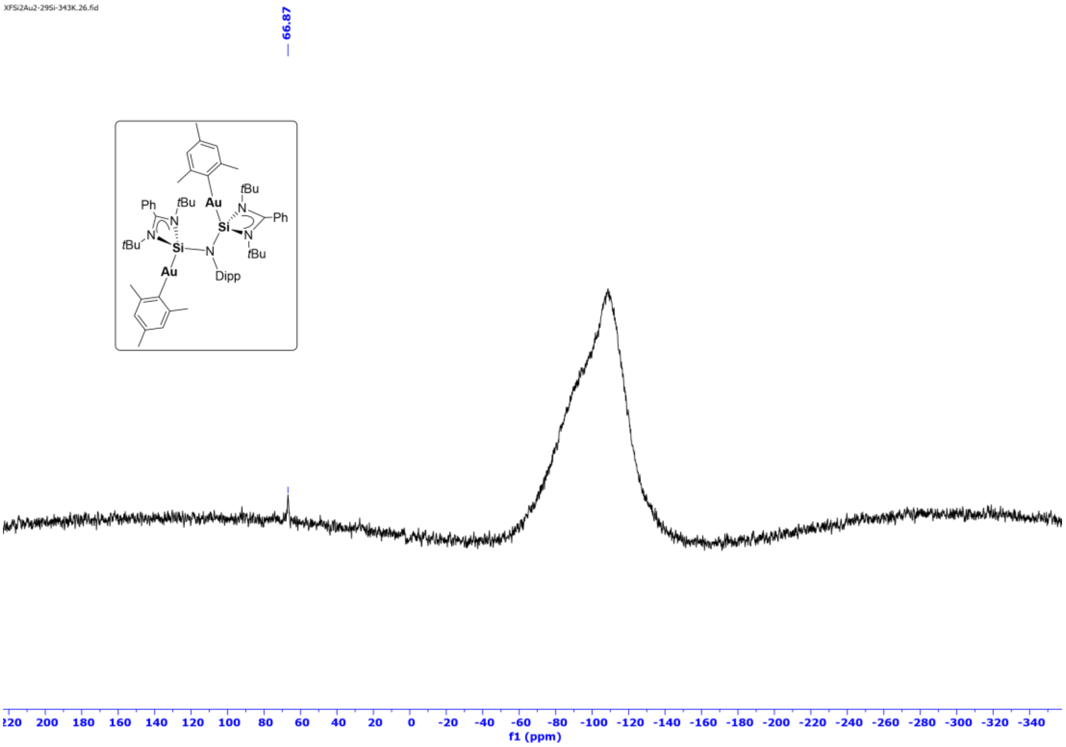

Figure S14. $^{29}Si\{^{1}H\}$ NMR spectrum of [LSi{Au(Mes)}−(NDipp)−{Au(Mes)}SiL] (**4**) in C_6D_6 measured at 343 K.

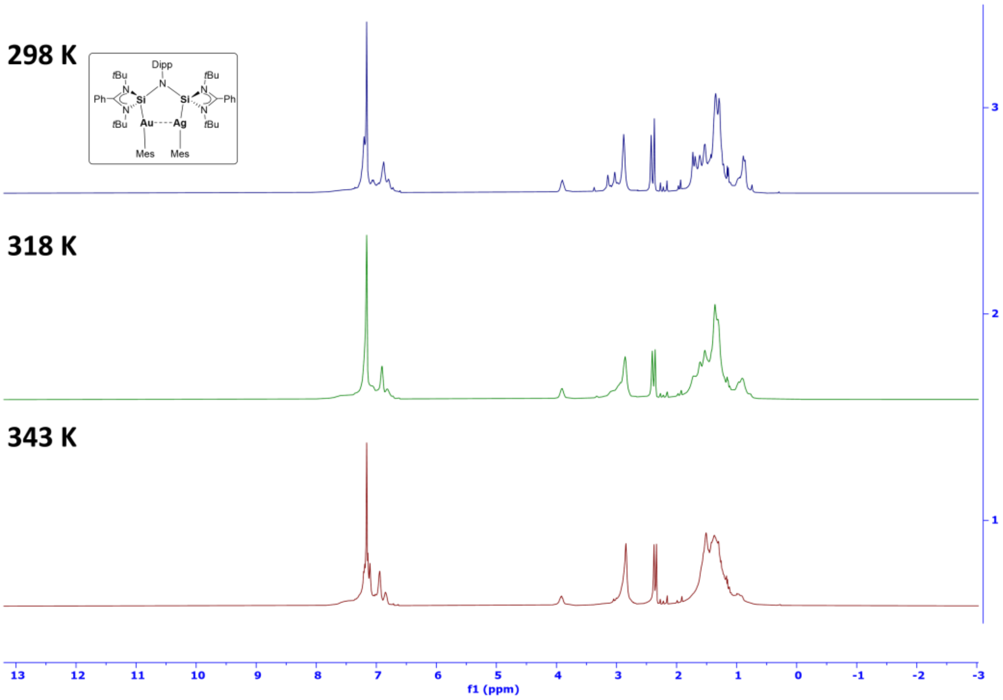

Figure S15. ^{1}H NMR spectrum of [LSi{Ag(Mes)}−(NDipp)−{Ag(Mes)}SiL] (**3**) in C_6D_6 measured from 298 K to 348 K.

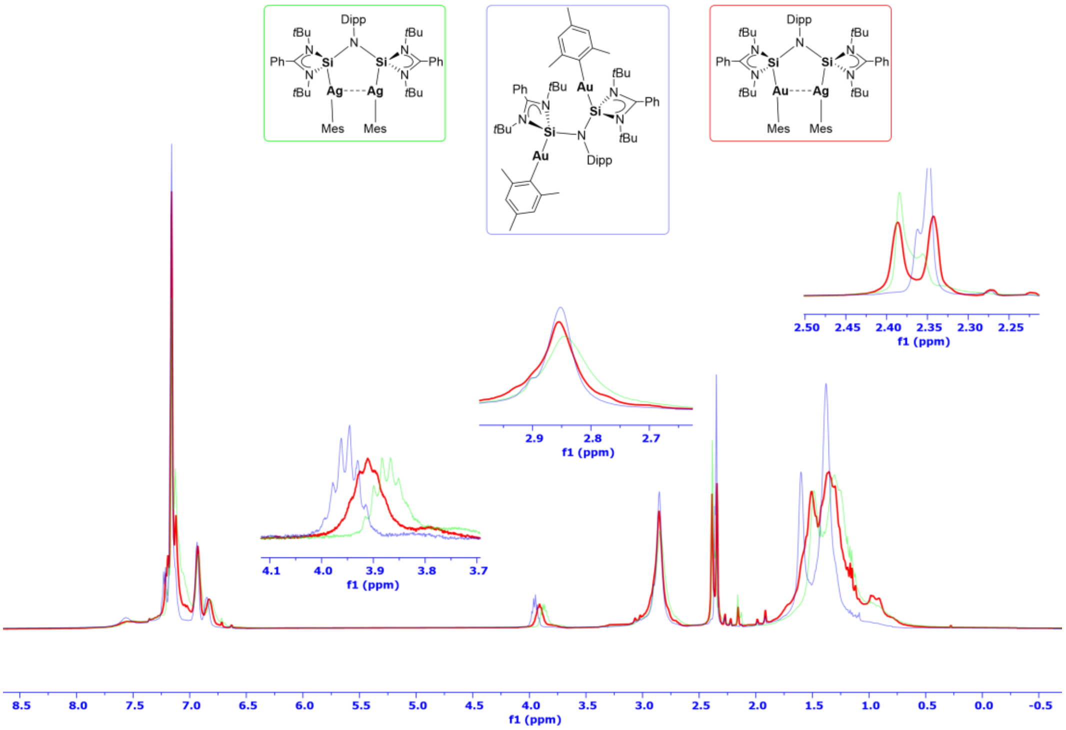

Figure S16. Overlapped ^{1}H NMR spectrum of complexes **3** (green), **4** (blue) and **5** (red) in C_6D_6 at 343 K.

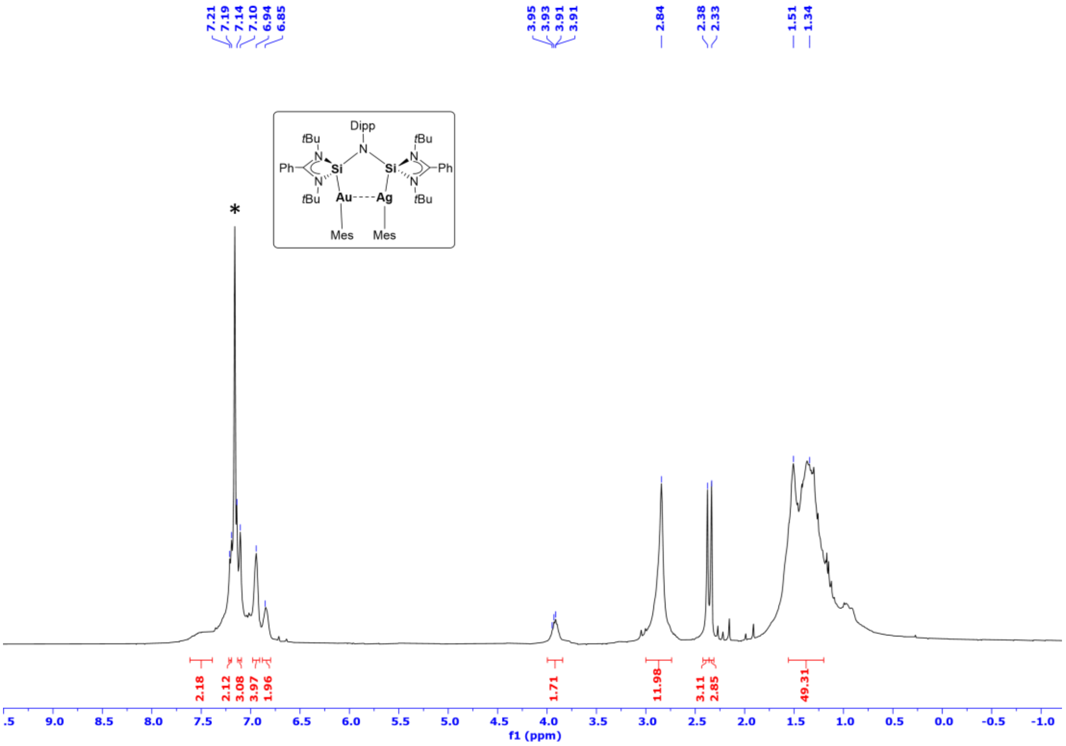

Figure S17. ^{1}H NMR spectrum of [LSi{Ag(Mes)}−(NDipp)−{Au(Mes)}SiL] (**5**) in C_6D_6 measured at 343 K.

*, residual protio solvent signal.

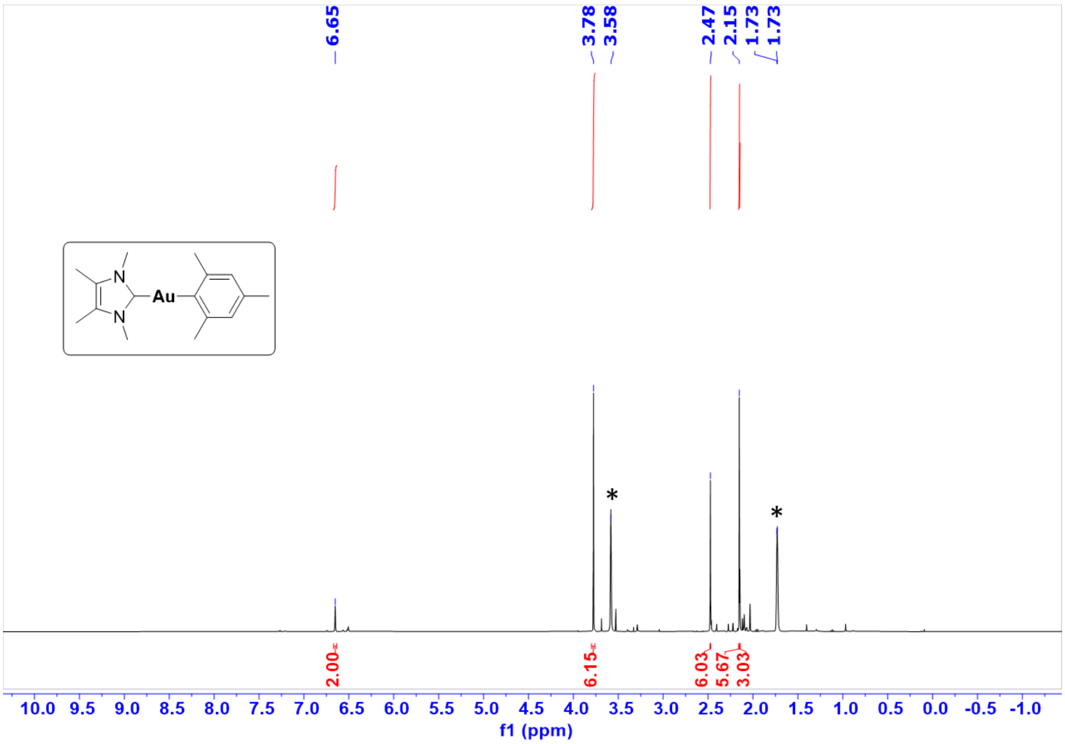

Figure S18. ^{1}H NMR spectrum of [ITMe{Au(Mes)}] (**6**) in THF-d_8 measured at 298 K. *, residual protio solvent signal.

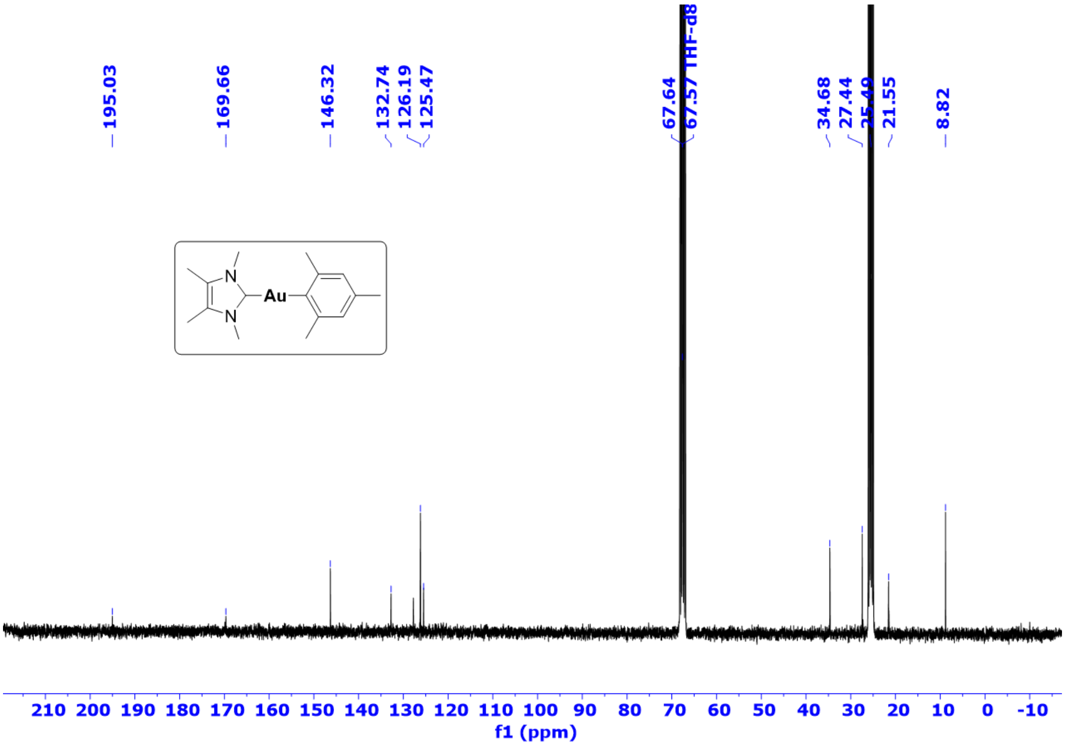

Figure S19. ^{13}C{^{1}H} NMR spectrum of [ITMe{Au(Mes)}] (**6**) in THF-d_8 measured at 298 K.

III. IR spectra

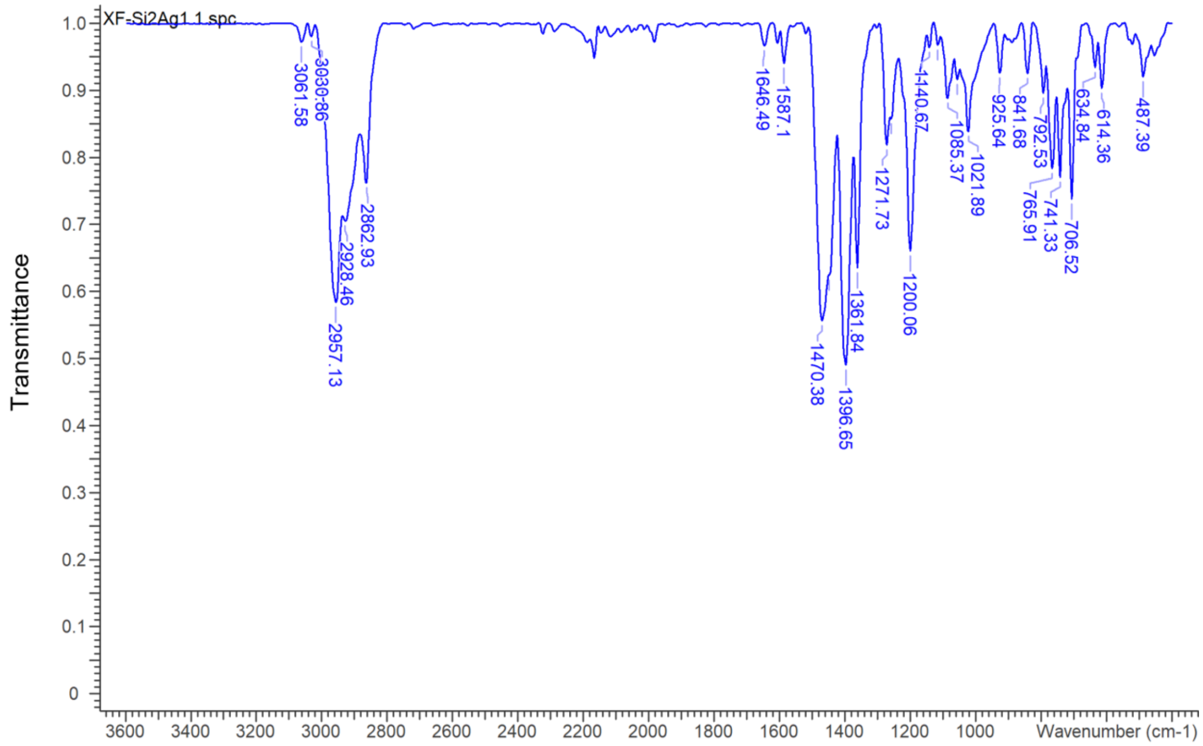

Figure S20. IR spectrum of complex **1**.

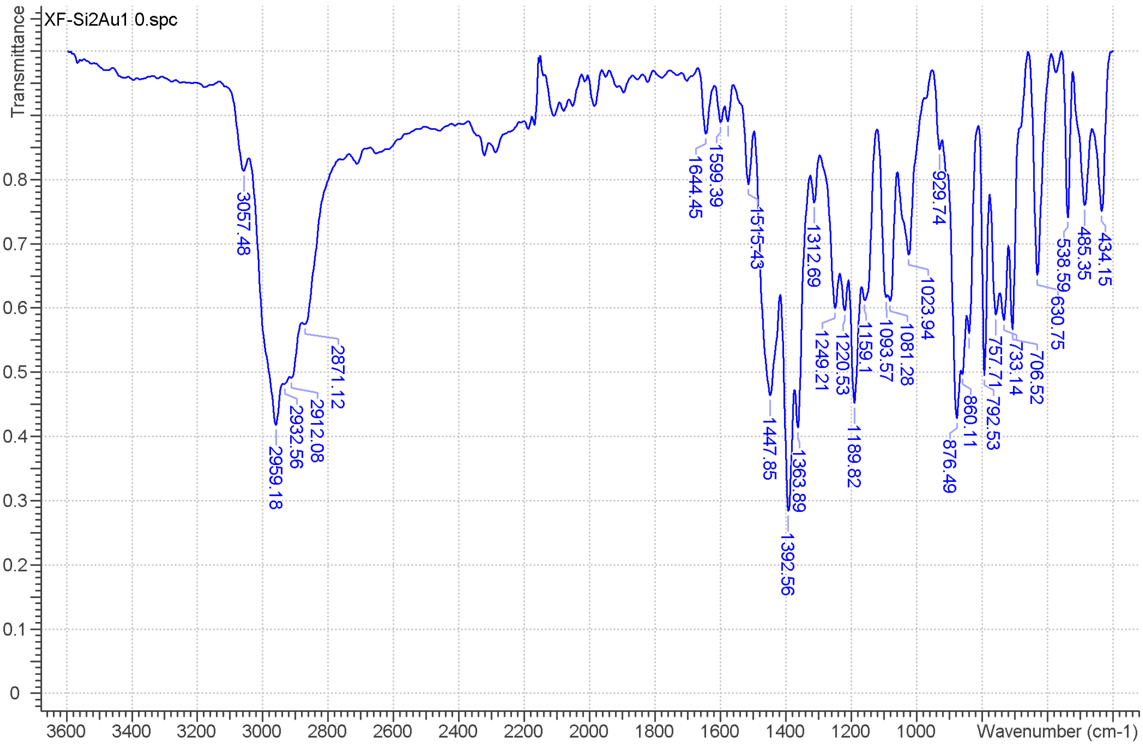

Figure S21. IR spectrum of complex **2**.

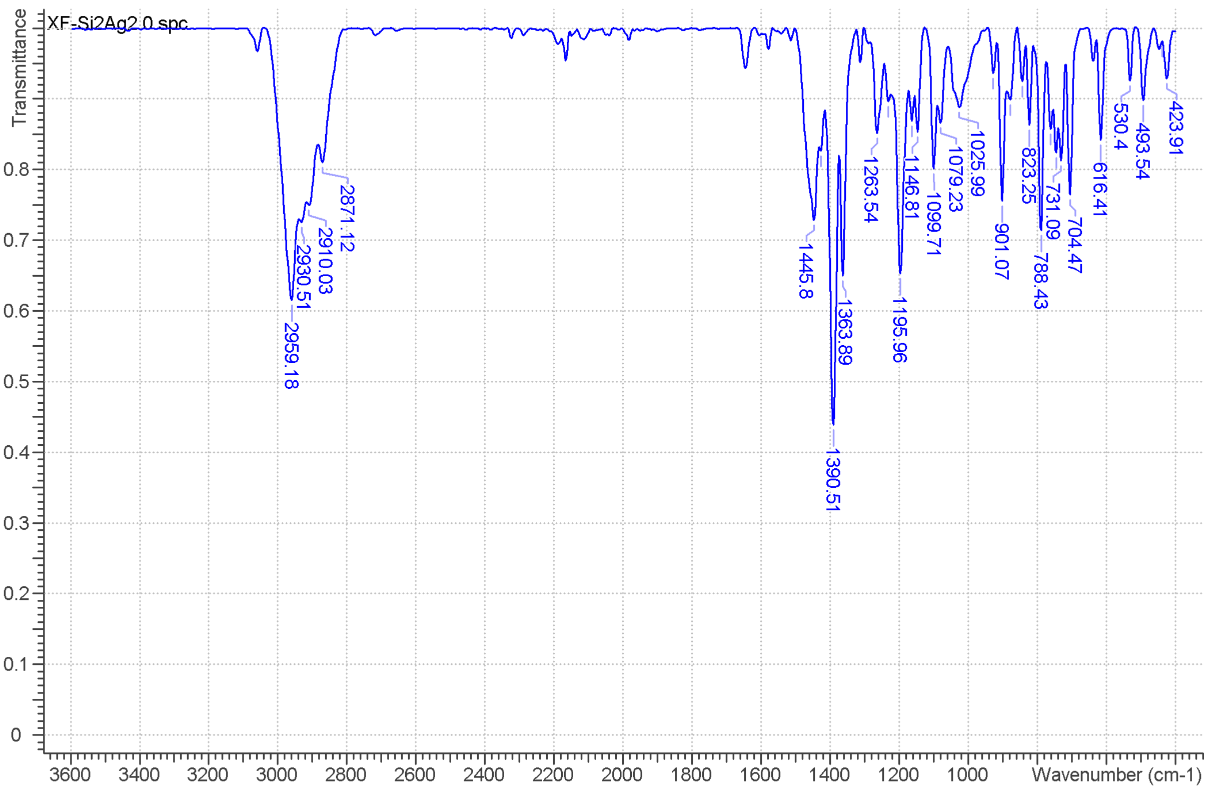

Figure S22. IR spectrum of complex **3**.

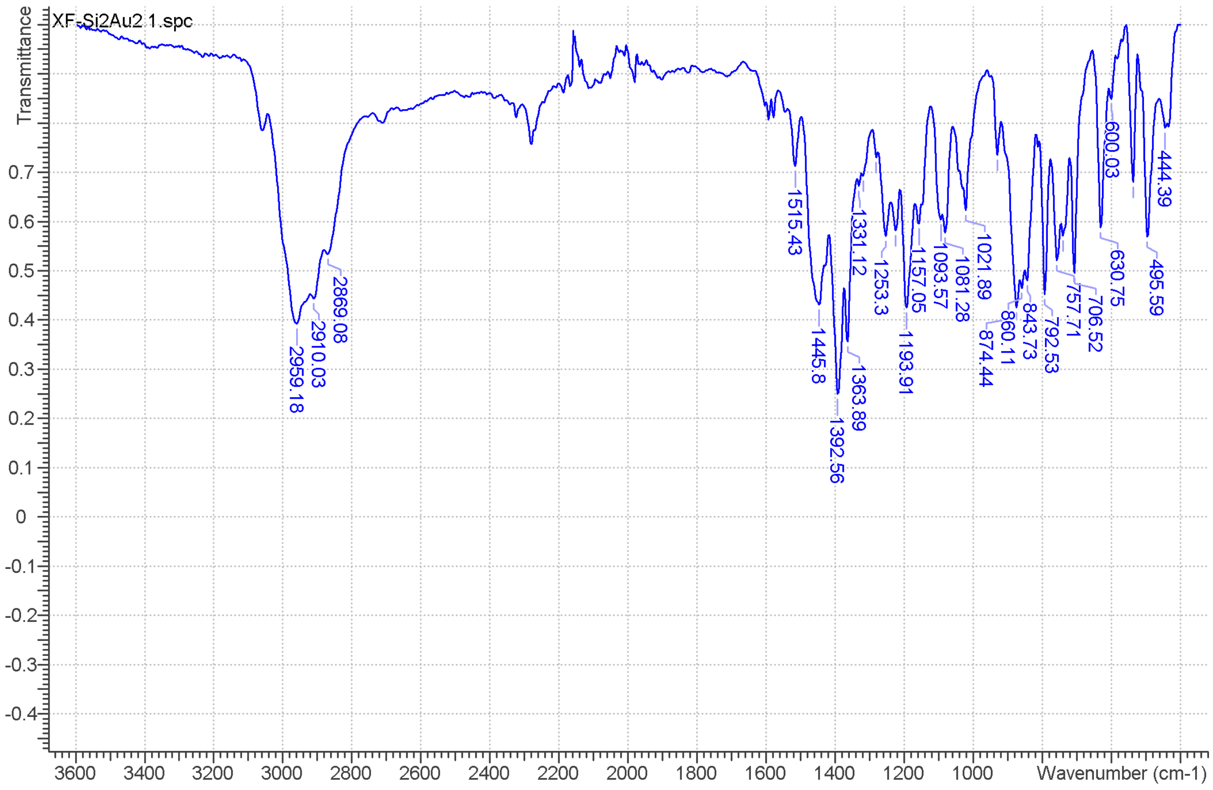

Figure S23. IR spectrum of complex **4**.

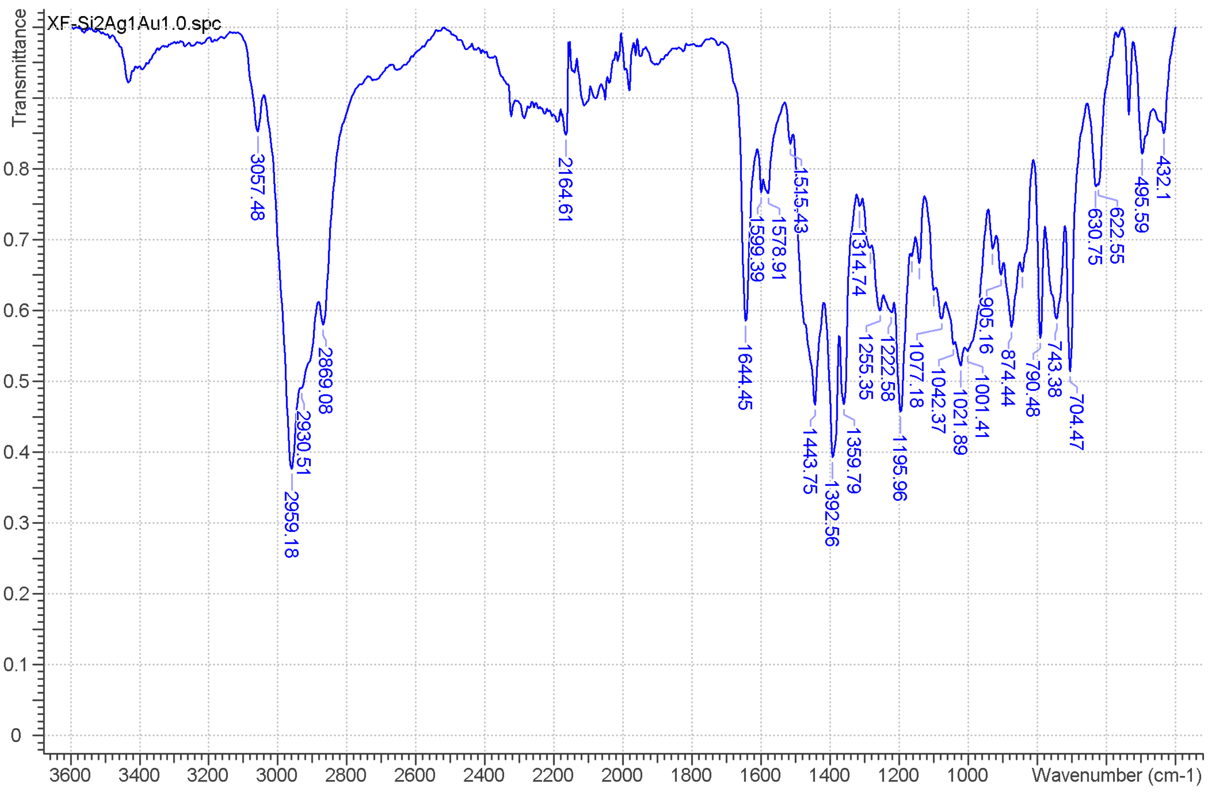

Figure S24. IR spectrum of complex **5**.

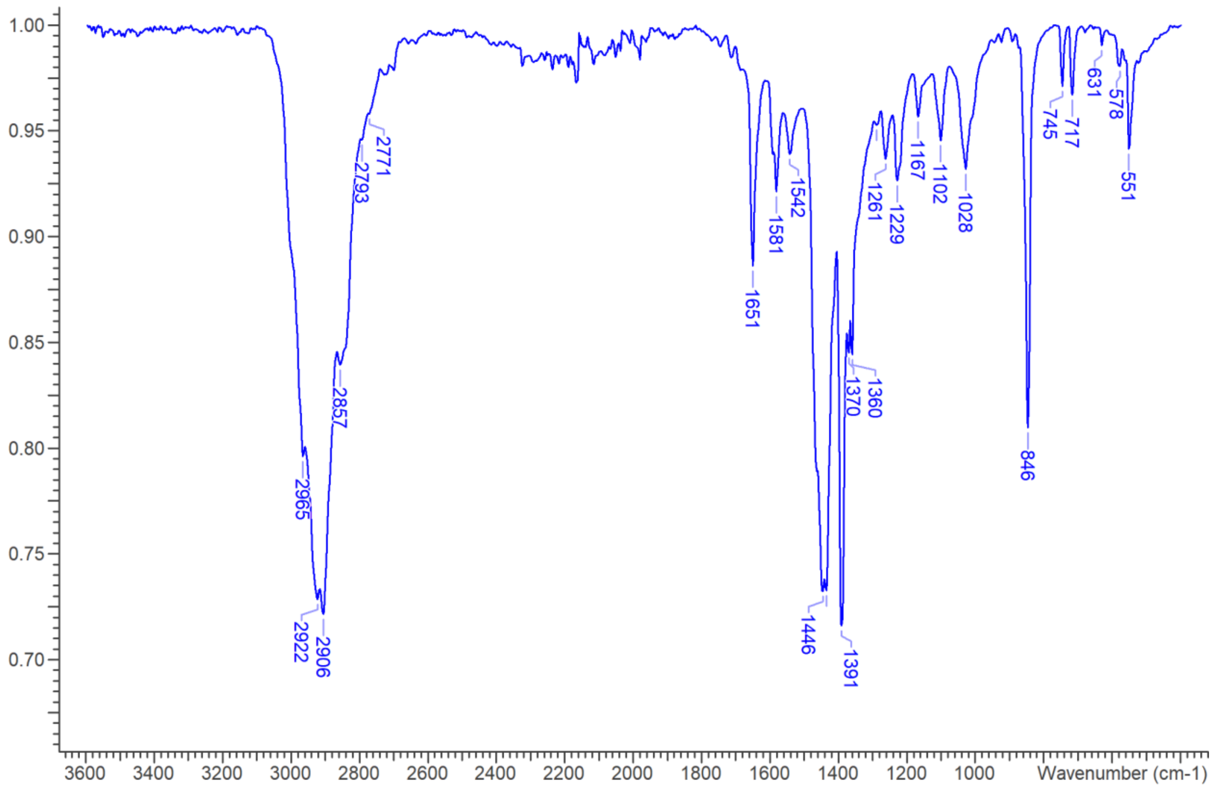

Figure S25 IR spectrum of complex **6**.

IV. X-ray crystallography

IV.1 General methods

Suitable crystals for the X-ray analysis of all compounds were obtained as described above. A suitable crystal was covered in mineral oil (Aldrich) and mounted on a glass fibre. The crystal was transferred directly to the cold stream of a STOE StadiVari (100 K or 110 K) diffractometer. All structures were solved by using the program SHELXS/T[4,5] and Olex2.[6] The remaining non-hydrogen atoms were located from successive difference Fourier map calculations. The refinements were carried out by using full-matrix least-squares techniques on F_o^2 by using the program SHELXL.[4,5] The H-atoms were introduced into the geometrically calculated positions (SHELXL procedures) unless otherwise stated and refined riding on the corresponding parent atoms. In each case, the locations of the largest peaks in the final difference Fourier map calculations, as well as the magnitude of the residual electron densities, were of no chemical significance. Specific comments for each data set are given below. Summary of the crystal data, data collection and refinement for compounds are given in Table S1.

Crystallographic data for the structures reported in this paper have been deposited with the Cambridge Crystallographic Data Centre as a supplementary publication no. CCDC 2393511-2393515 and 2448315-2448316. Copies of the data can be obtained free of charge on application to CCDC, 12 Union Road, Cambridge CB21EZ, UK (fax: (+(44)1223-336-033; email: deposit@ccdc.cam.ac.uk).

The following special comments were applied to the models of the structures:

In the structure of complex **2**, one co-crystallized C_6D_6 molecule (C52-C57) is disordered over two positions with an occupancy ratio of 0.77/0.23. One half co-crystallized C_6D_6 molecule (C58-C60) is disordered over two positions with an occupancy ratio of 0.58/0.42.

In the structure of **3**, one *t*Bu group (C20-C23) is disordered over two positions with an occupancy ratio of 0.70/0.30.

For the refinement of the structure of **4**, solvent mask was applied to eliminate the co-crystallized C_6D_6 molecules.

In the structure of **4'**, one *t*Bu group (C14-C17) is disordered over two positions with an occupancy ratio of 0.64/0.36.

In the structure of **5**, EADP was used for the Ag and Au atoms to restrain isotropic thermal parameters. One *t*Bu group (C27-C30) is disordered over two positions with an occupancy ratio of 0.66/0.34.

IV.2 Summary of crystal data

Table S1. Crystal data and structure refinement for compounds **1-6.**

**Compound**	**1**	**2**	**3**	**4**	**4'**	**5**	**6**
Formula	$C_{51}H_{74}AgN_5Si_2$ (C_7H_8)	$C_{51}H_{74}AuN_5Si_2$ 1.5(C_6D_6)	$C_{60}H_{85}Ag_2N_5Si_2$	$C_{60}H_{85}Au_2N_5Si_2$ (sol)	$C_{60}H_{85}Ag_2N_5Si_2$ 4(C_6D_6)	$C_{84}H_{85}AgAuD_{24}N_5Si_2$	C16H23AuN2
$D_{calc.}$/ g cm^{-3}	1.197	1.311	1.324	1. 419	1.451	1.368	1.870
μ/mm^{-1}	0.441	2.638	4.108	3.899	3.929	2.252	9.398
Formula Weight	1013.33	1136.51	1148.24	1638.87	1663.02	1573.92	440.33
Colour	yellow	yellow	colourless	orange	yellow	yellow	colourless
Shape	plate-shaped	block-shaped	block-shaped	plate-shaped	rod-shaped	plate-shaped	block-shaped
T/K	100	110	150	110	100	110	100
Crystal System	triclinic	triclinic	monoclinic	monoclinic	monoclinic	monoclinic	triclinic
Space Group	P-1	P-1	$P2_1/c$	$C2/c$	$C2/c$	$C2/c$	P-1
a/Å	14.0034(11)	12.4819(19)	13.0057(10)	39.2308(9)	23.4582(17)	23.348(6)	7.3250(3)
b/Å	14.5552(11)	15.9578(12)	25.263(2)	18.5805(3)	13.7319(8)	13.839(4)	8.2648(4)
c/Å	14.8701(11)	16.7970(14)	17.7227(15)	21.8747(5)	25.7140(19)	25.682(9)	13.2970(6)
α/°	71.802(6)	71.514(6)	90	90	90	90	92.910(4)
β/°	77.731(6)	69.239(10)	98.425(6)	105.850(2)	113.237(5)	112.95(2)	103.140(4)
γ/°	88.727(6)	70.868(10)	90	90	90	90	92.282(4)
V/Å^3	2810.3(4)	2878.2(6)	5760.2(8)	15338.8(6)	7611.2(9)	7641(4)	781.82(6)
Z	2	2	4	8	4	4	2
Z'	1	1	1	1	0.5	0.5	1
Wavelength/ Å	0.71073	0.71073	1.34143	0.71073	0.71073	0.71073	0.71073
Radiation type	MoK_α	MoK_α	GaK_α	MoK_α	Mo K[illegible]	MoK_α	Mo K[illegible]
Θ_{min}/°	2.853	1.799	2.988	1.647	2.186	1.722	2.848
Θ_{max}/°	29.229	25.999	64.561	25.249	30.308	25.998	29.403

Measured Refl's.	43558	28043	38672	63719	28983	21686	15014
Indep't Refl's	13279	11285	13889	13867	10128	7489	15014
Refl's I≥2 σ(I)	10543	10182	11259	11995	8262	5399	13862
R_{int}	0.0377	0.0298	0.0204	0.0496	0.0264	0.0454	
Parameters	667	691	684	644	472	475	180
Restraints	428	229	160	0	106	106	0
Largest Peak	0.589	1.094	0.404	3.607	1.381	1.278	1.792
Deepest Hole	-0.460	-0.760	-0.447	-1.236	-1.319	-0.714	-1.976
GooF	1.056	1.038	0.952	1.068	1.046	1.017	1.142
wR_2 (all data)	0.0864	0.0736	0.0576	0.1486	0.0989	0.1026	0.0780
wR_2	0.0833	0.0713	0.0561	0.1433	0.0941	0.0898	0.0772
R_1 (all data)	0.0461	0.0353	0.0315	0.0678	0.0495	0.0766	0.0309
R_1	0.0329	0.0294	0.0232	0.0596	0.0365	0.0436	0.0285

IV.2 Crystal structures

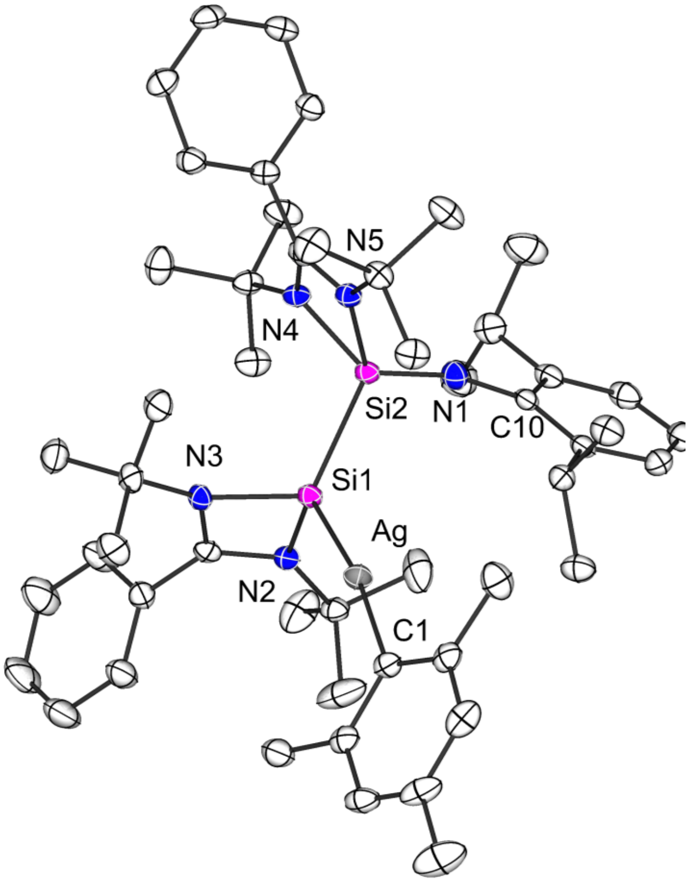

Figure S26. Molecular structure of the complex **1** in the solid state with thermal ellipsoids at 40% level. Selected bond distances [Å] and angles [°]: Ag–Si1 2.3847(5), Ag–C1 2.122(2), Si1–Si2 2.3593(7), Si2–N1 1.576(2), N1–C10 1.368(2); Si1–Ag–C1 169.84(5), Ag–Si1–Si2 125.16(2), Si1–Si2–N1 117.28(6), Si2–N1–C10 164.81(14).

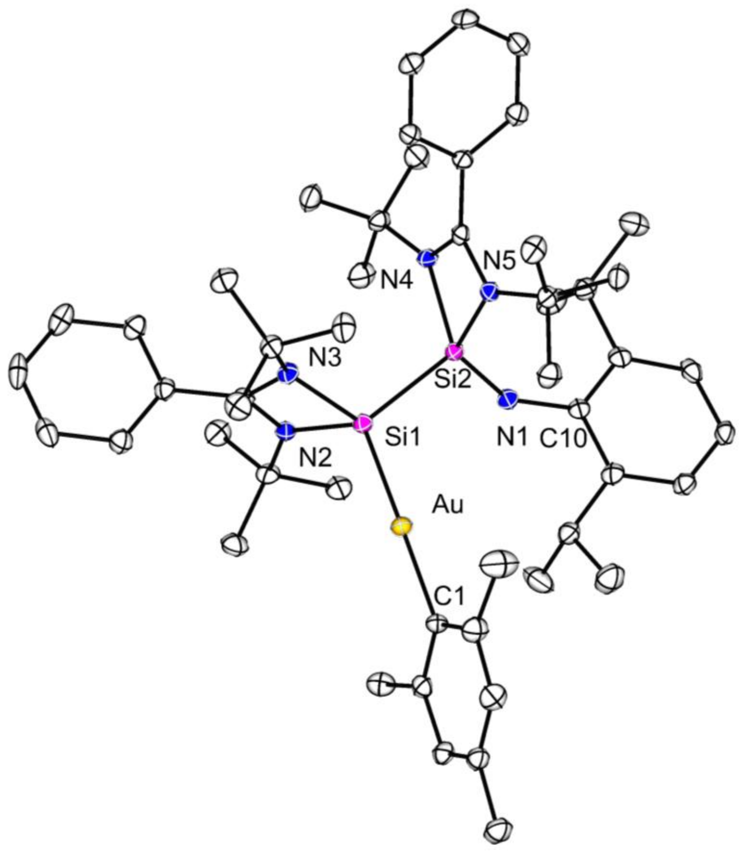

Figure S27. Molecular structure of the complex **2** in the solid state with thermal ellipsoids at 40% level. Selected bond distances [Å] and angles [°]: Au–Si1 2.3006(8), Au–C1 2.079(3), Si1–Si2 2.3514(11), Si2–N1 1.597(3), N1–C10 1.385(4); Si1–Au–C1 175.21(9), Au–Si1–Si2 114.49(4), Si1–Si2–N1 109.75(10), Si2–N1–C10 143.2(2).

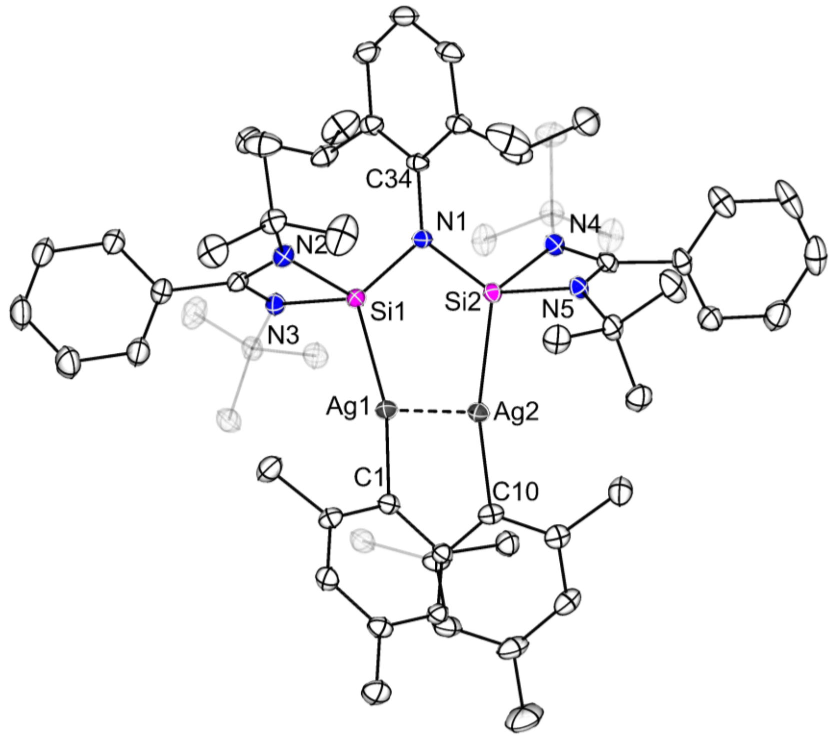

Figure S28. Molecular structure of the complex **3** in the solid state with thermal ellipsoids at 40% level. Selected bond distances [Å] and angles [°]: Ag1…Ag2 3.1235(3), Ag1–Si1 2.3993(5), Ag2–Si2 2.4191(5), Ag1–C1 2.135(2), Ag1–C10 2.147(2), Si1–N1 1.7741(13), Si2–N1 1.7796(14), N1–C34 1.471(2); Si1–Ag1–C1 164.11(5), Si2–Ag2–C2 165.52(5), Si1–N1–Si2 104.35(7).

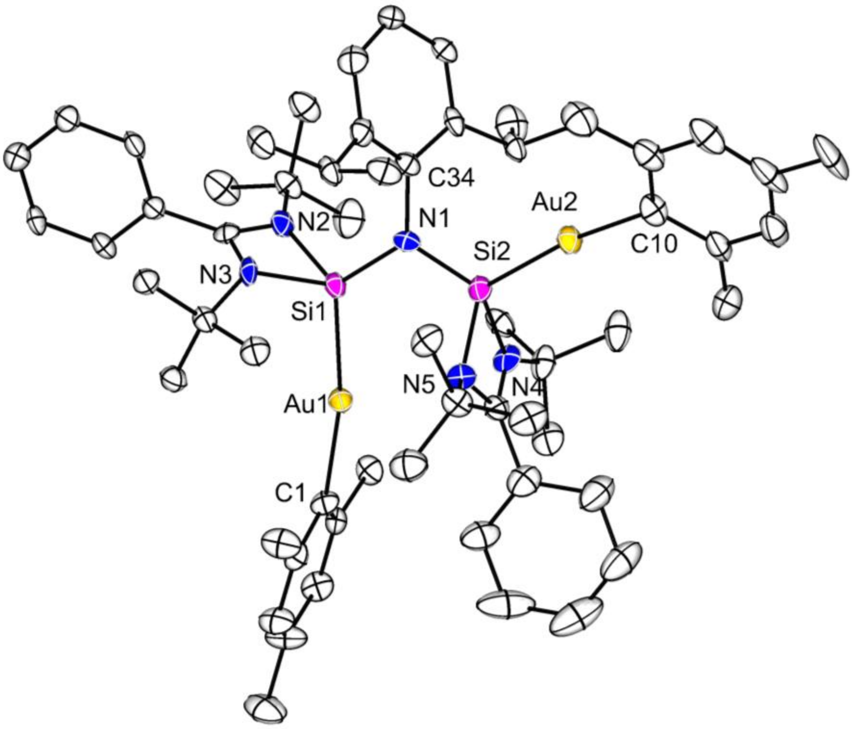

Figure S29 Molecular structure of the complex **4** in the solid state with thermal ellipsoids at 40% level. Selected bond distances [Å] and angles [°]: Au1–Si1 2.318(2), Au2–Si2 2.307(2), Au1–C1 2.096(8), Au1–C10 2.072(8), Si1–N1 1.778(7), Si2–N1 1.759(7), N1–C34 1.478(10); Si1–Au1–C1 169.3(2), Si2–Au2–C10 171.9(3), Si1–N1–Si2 120.1(4).

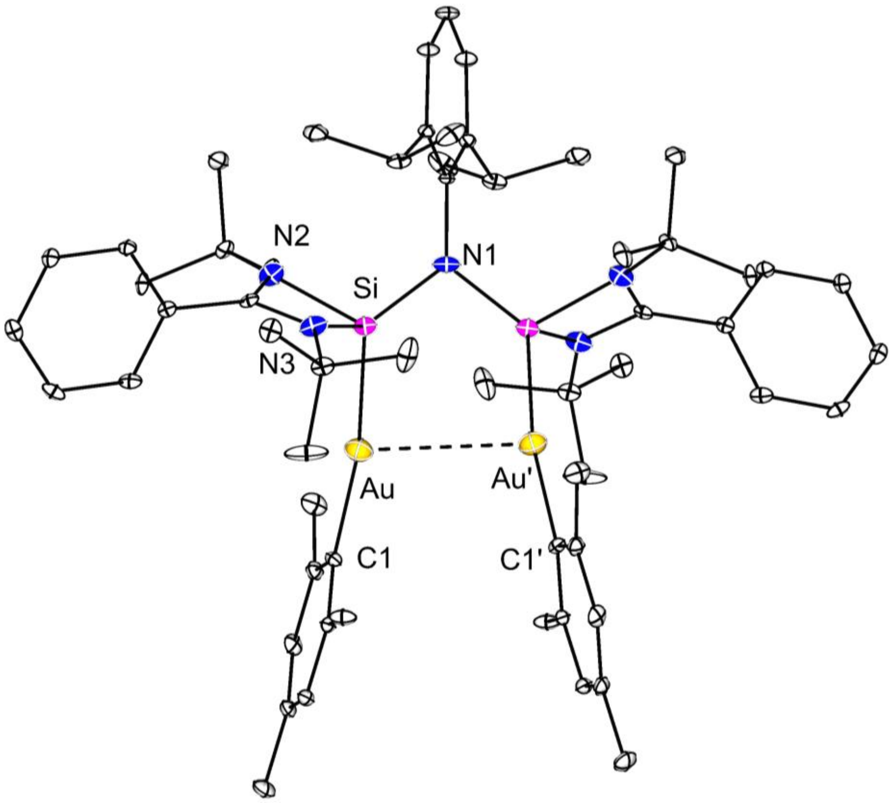

Figure S30 Molecular structure of the complex **4'** in the solid state with thermal ellipsoids at 40% level. Selected bond distances [Å] and angles [°]: Au…Au**'** 3.4545(4), Au–Si 2.3072(10), Au–C1 2.066(4), Si1–N1 1.764(3); Si–Au–C1 170.56(11), Si–N1–Si' 105.0(2).

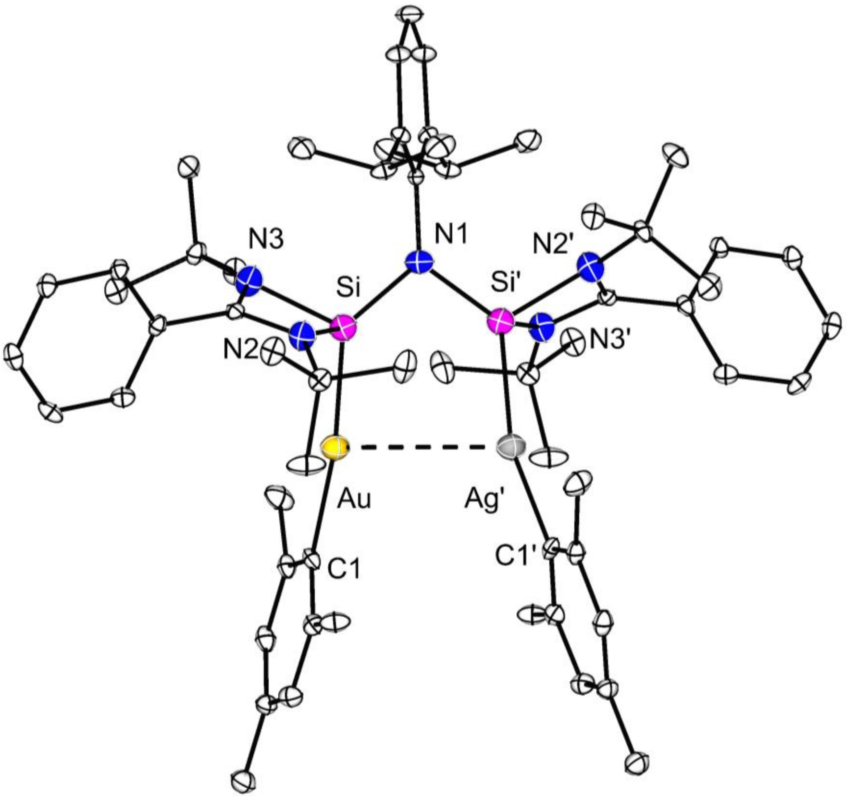

Figure S31 Molecular structure of the complex **5** in the solid state with thermal ellipsoids at 40% level. Selected bond distances [Å] and angles [°]: Si–Ag‘ 2.406(9), Si–Au 2.322(5), Au–C1 2.156(6), Ag’–C1’ 2.099(10); Si–Au–C1 172.0(2), Si’–Ag‘–C1‘ 165.0(4).

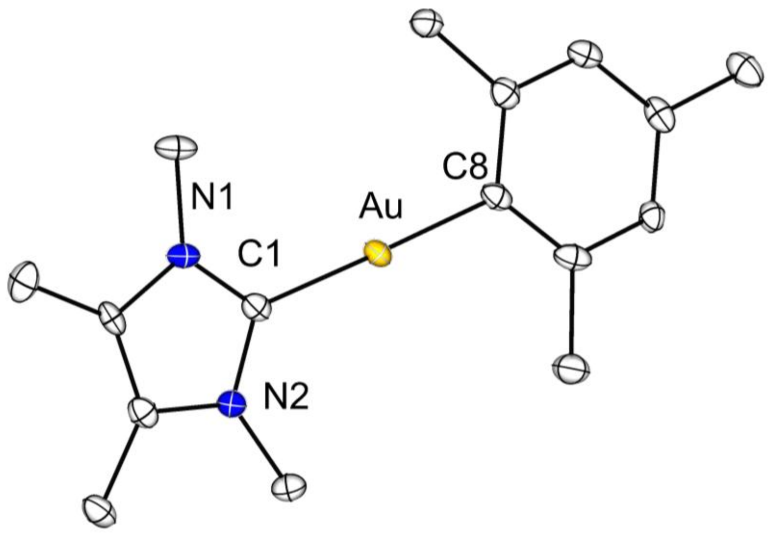

Figure S32 Molecular structure of the complex **6** in the solid state with thermal ellipsoids at 40% level. Selected bond distances [Å] and angles [°]: C1–Au 2.042(5), C8–Au 2.046(5); C1–Au–C8 178.6(2).

V. Calculations

Table S2. Results of the DFT calculations: important structural (in Å or °) and energy data (in kJ mol^{-1}), population analyses based on occupation numbers and local force constant values (given in mdyn $Å^{-1}$).

	**3**<br>M=M'=Ag	**4'**<br>M=M'=Au	**5**<br>M=Ag, M'=Au
E(cis)-E(trans)	-5.5	-3.1	-11.7
r(M-M)	3.169	3.281	3.333
r(M-Si/M'-Si)	2.405	2.368	2.403/2.371
r(M-C/M'-C)	2.123	2.102	2.121/2.105
<(Si-M-C)	168.4/174.0	173.5/174.0	173.4/173.7
<(Si-N-Si)	101.5	101.8	101.4
<(M-Si…Si-M') Torsion	40.1°	43.0°	43.1°
AIM			
ρ	0.0186	0.0199	0.0163
BE(AIM)(M-M')/kJ mol^{-1}	19.5	15.4	
PABOON			
SEN(M-M')	0.05	0.02	0.02
SEN(M-Si/M'-Si)	0.40/0.34	0.50/0.53	0.40/0.49
SEN(M-C/M'-C)	0.40/0.44	0.38/0.39	0.43/0.40
Q(M/M')	0.09/0.09	0.08/0.08	0.06/0.08
Q(2M-Mes/2M'-Mes)	-0.50	-0.69	-0.62
local force constant/mdyn $Å^{-1}$	0.077	0.144	0.091

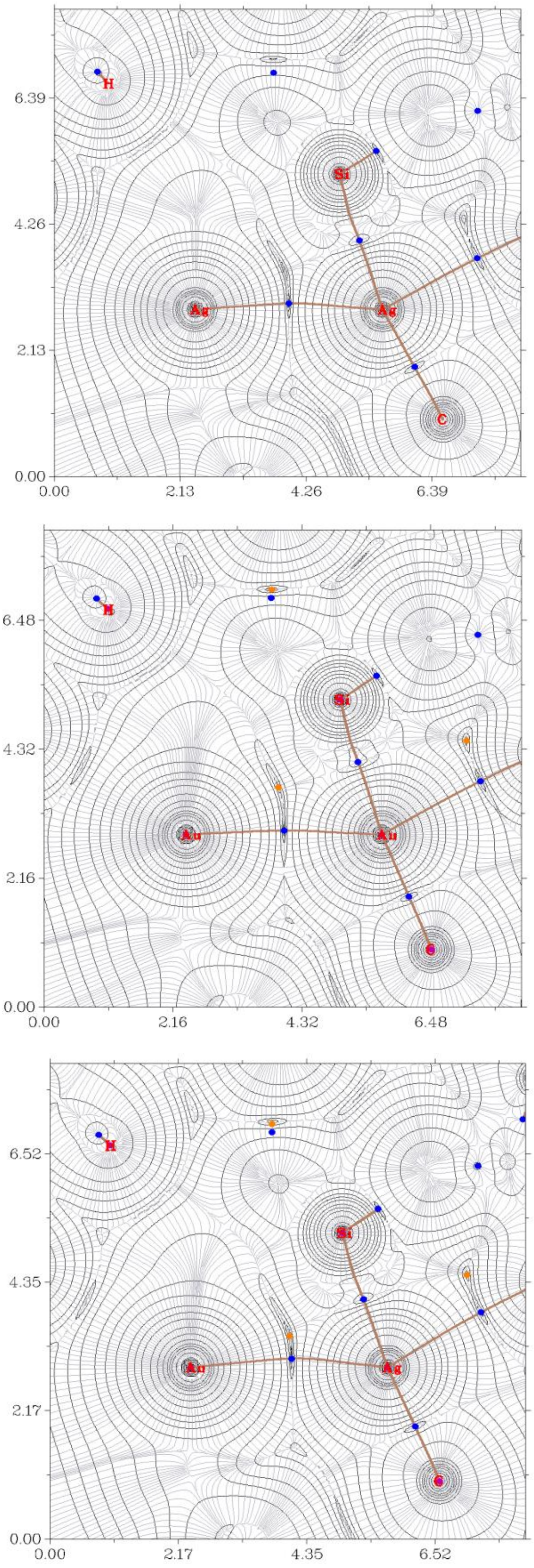

Figure S33. Electronic density and gradient plots of **3**, **4’** and **5**: bond paths and bond critical points (in blue).

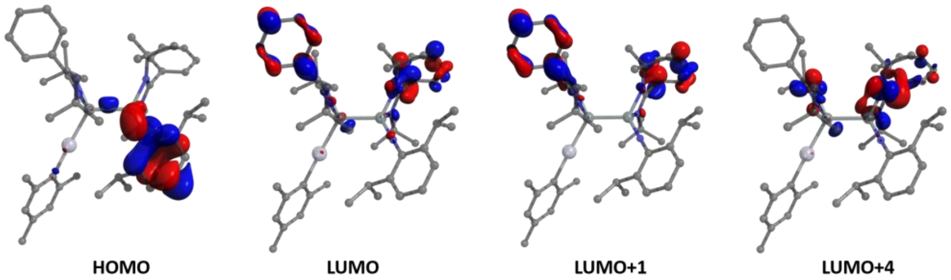

Figure S34. Selected MOs of complex **1**.

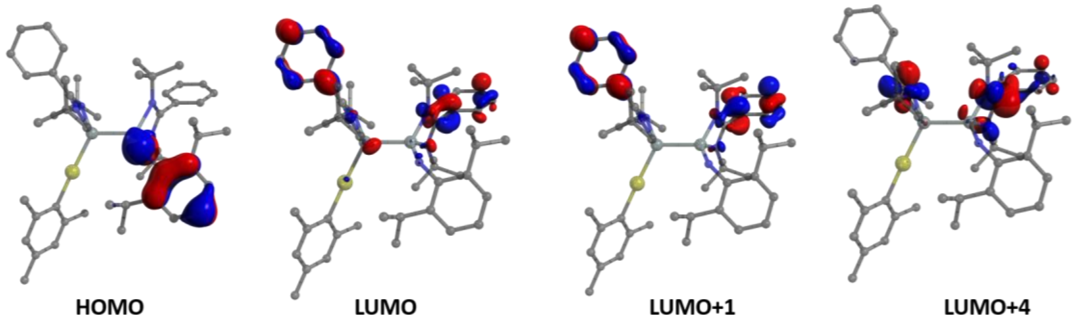

Figure S35. Selected MOs of complex **2**.

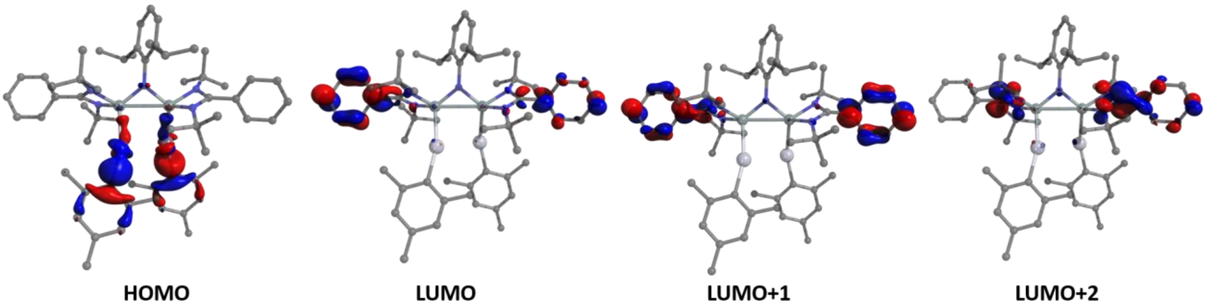

Figure S36. Selected MOs of complex **3**.

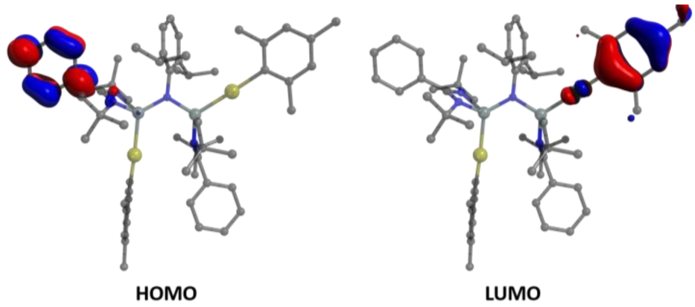

Figure S37. Selected MOs of complex **4**.

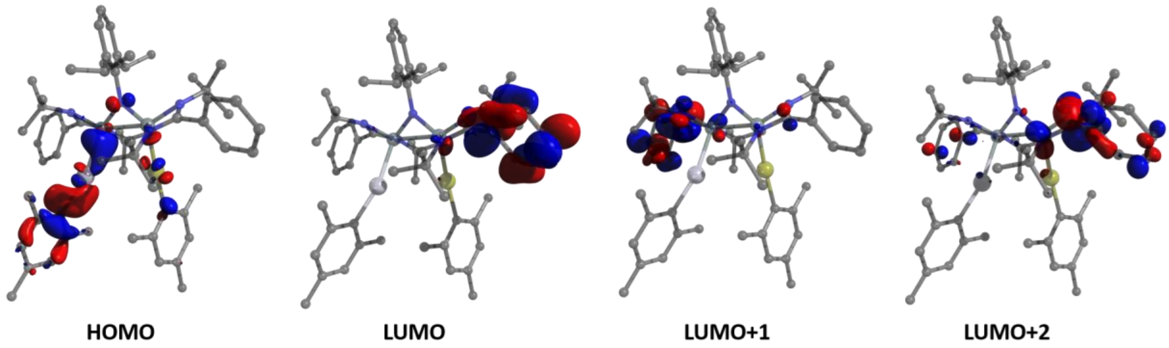

Figure S38. Selected MOs of complex **5**.

UV-Vis – Calculated absorption maxima of all complexes

Complex 1 (Ag1)

Excited State 1: Singlet-A 2.5212 eV 491.77 nm f=0.0280 <S**2>=0.000

231 -> 232 0.63800

Excited State 2: Singlet-A 2.7643 eV 448.52 nm f=0.0213 <S**2>=0.000

231 -> 233 0.68686

Excited State 3: Singlet-A 2.9307 eV 423.05 nm f=0.0141 <S**2>=0.000

231 -> 236 0.62311

Complex 2 (Au1)

Excited State 1: Singlet-A 2.6382 eV 469.95 nm f=0.0038 <S**2>=0.000

231 -> 232 0.63427

Excited State 2: Singlet-A 2.9563 eV 419.39 nm f=0.0034 <S**2>=0.000

231 -> 233 0.67080

Excited State 3: Singlet-A 3.1243 eV 396.84 nm f=0.0183 <S**2>=0.000

231 -> 236 0.63970

Complex 3 (Ag2)

Excited State 1: Singlet-A 3.1896 eV 388.71 nm f=0.0042 <S**2>=0.000

273 -> 274 -0.46076

273 -> 275 0.22904

273 -> 276 0.46090

Complex 4 (Au2 – cis)

Excited State 1: Singlet-A 3.5620 eV 348.07 nm f=0.0448 <S**2>=0.000

272 -> 276 0.61152

Complex 4 (Au2 trans)

Excited State 1: Singlet-A 3.8254 eV 324.11 nm f=0.0031 <S**2>=0.000

273 -> 274 0.70402

Complex 5 (Ag1Au1)

Excited State 1: Singlet-A 3.2202 eV 385.02 nm f=0.0416 <S**2>=0.000

273 -> 274 0.55232

273 -> 275 0.23223

273 -> 276 0.30919

Excited State 2: Singlet-A 3.3777 eV 367.06 nm f=0.0088 <S**2>=0.000

273 -> 274 -0.14613

273 -> 275 0.64377

273 -> 276 -0.22105

VI. UV-vis spectra

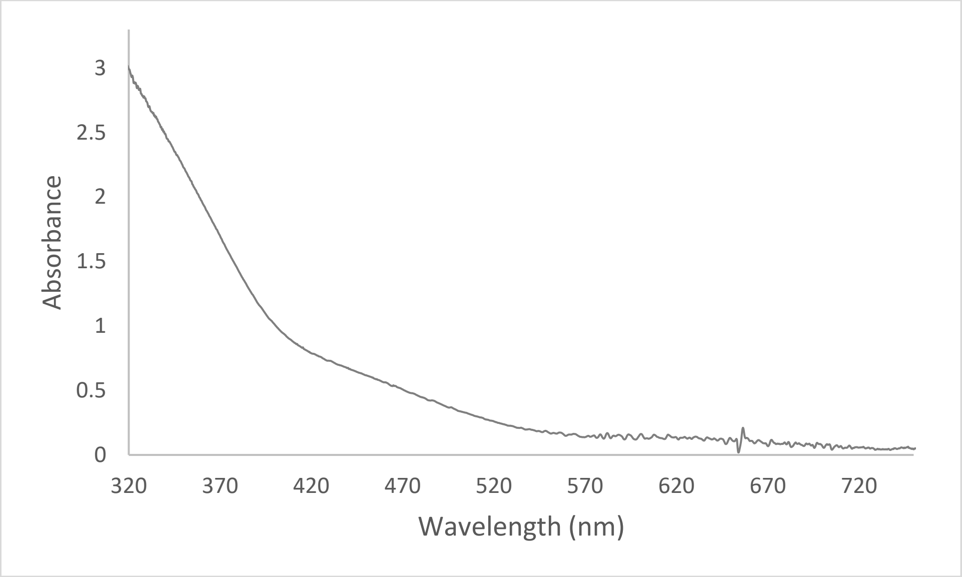

Figure S39. UV-vis spectrum of complex **1** in toluene.

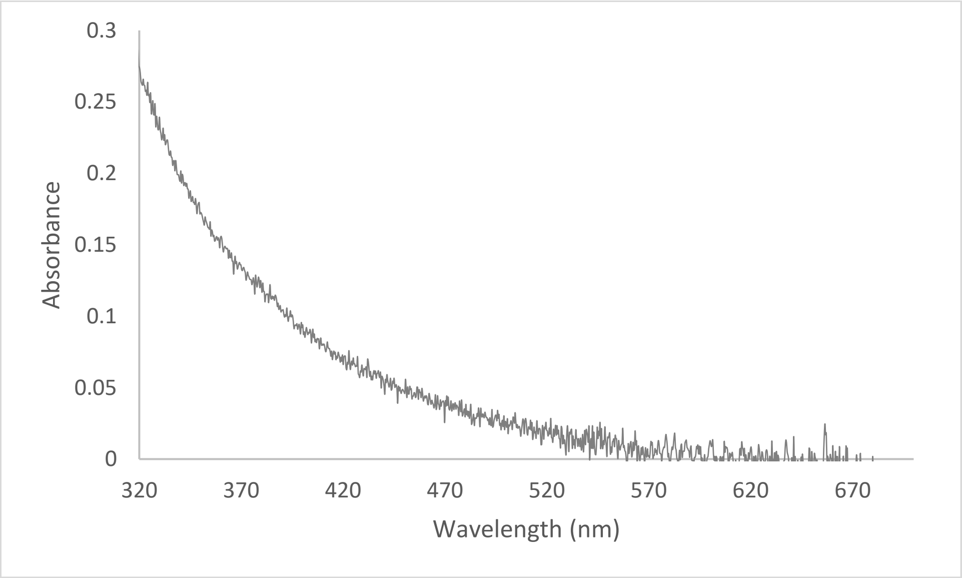

Figure S40. UV-vis spectrum of complex **2** in toluene.

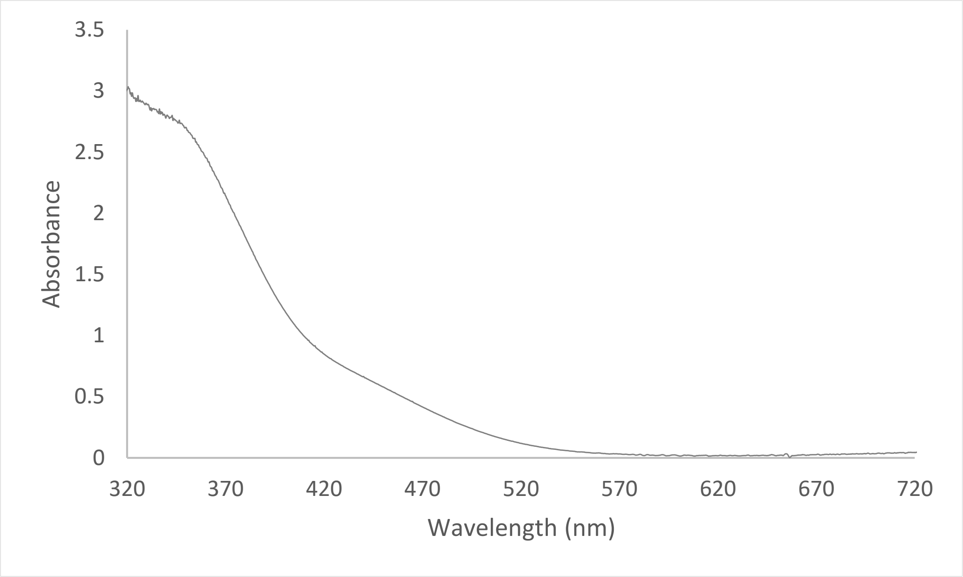

Figure S41. UV-vis spectrum of complex **3** in toluene.

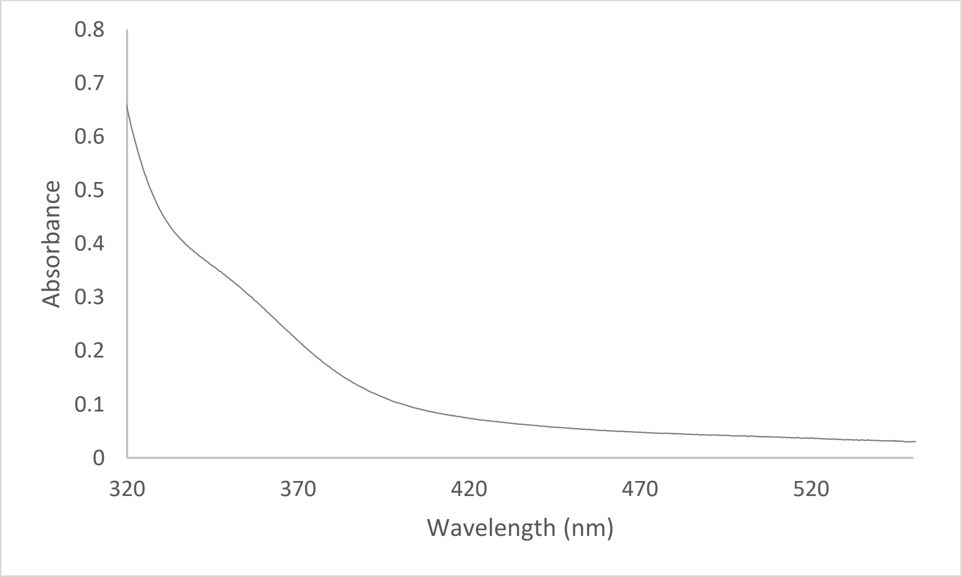

Figure S42. UV-vis spectrum of complex **4** in toluene.

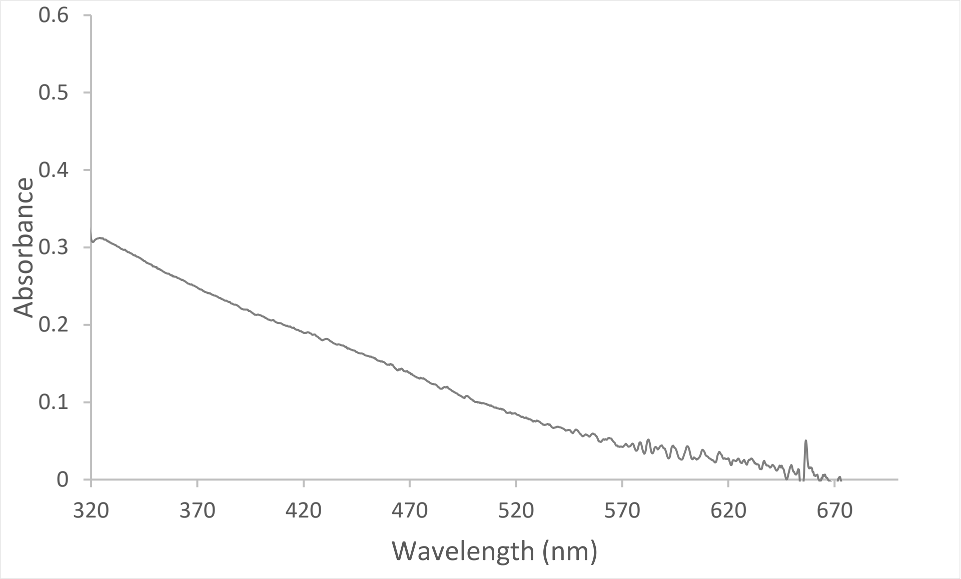

Figure S43. UV-vis spectrum of complex **5** in toluene.

VII. References

[1] R. Yadav, X. Sun, R. Köppe, M. T. Gamer, F. Weigend and P. W. Roesky, *Angew. Chem.Int. Ed*. **2022**, *61*, e20221111.

[2] E. M. Meyer, S. Gambarotta, C. Floriani, A. Chiesi-Villa and C. Guastini, *Organometallics*, **1989**, *8*, 1067-1079.

[3] G. Siddiqi, V. Mougela and C. Copéret, *Dalton Trans*., **2015**, *44*, 14349-14353.

[4] G. Sheldrick, *Acta Cryst. A* **2008**, *64*, 112-122.

[5] G. Sheldrick, *Acta Cryst. C* **2015**, *A71*, 3-8.

[6] O. V; Dolomanov, L, J, Bourhis, R. J. Gildea, J. A. K. Howard, H. Puschmann, *J. Appl. Crystallogr*. **2009**, *42*, 339-341.
